# Early or late distractions hurt working memory differently depending on how long you look

**DOI:** 10.1038/s41598-025-18699-z

**Published:** 2025-10-09

**Authors:** Guofang Ren, Ruyi Liu, Lijing Guo, Penglan Liu, Dan Nie, Jinru Chen, Chaoxiong Ye

**Affiliations:** 1 School of Education, Anyang Normal University, Anyang, 455000 China; 2 Department of Psychology, University of Jyvaskyla, 40014 Jyväskylä, Finland; 3 Institute of Brain and Psychological Sciences, Sichuan Normal University, Chengdu, 610066 China

**Keywords:** Visual short-term memory, Distractor filtering, Stimulus presentation duration, Encoding stage, Delay stage, Psychology, Human behaviour

## Abstract

Visual Working Memory (VWM) is essential for temporarily retaining goal-relevant visual information, yet its limited capacity renders it vulnerable to distraction. While previous studies have examined the effects of distractors occurring during encoding or maintenance, it remains unclear how stimulus presentation duration modulates these effects and whether different types or quantities of distractors exert similar costs. Across three experiments, we systematically investigated how distraction timing, distractor content, and distraction load interact with encoding duration to influence VWM performance. In Experiment 1, participants performed continuous recall and change detection tasks under four distraction conditions (no-, encoding-, delay-, and full-distraction) and two encoding durations (short: 200 ms; long: 1000 ms). Encoding-stage distractions impaired performance only in the short-duration, high-precision task, whereas delay-stage distractions consistently disrupted memory regardless of duration or task type. Experiment 2 manipulated distractor-target similarity (same-category vs. different-category distractors) and revealed that homogeneous distractors exerted stronger disruption, particularly when presented during the delay period. In contrast, heterogeneous distractors could be effectively suppressed when sufficient encoding time allowed for robust consolidation. Experiment 3 examined perceptual load (low vs. high) and showed that increasing distractor quantity did not amplify interference, suggesting that once memory consolidation is complete, delay-stage distractions disrupt VWM representations regardless of distractor load. Together, these findings reveal an asymmetry in how VWM handles distractions at different processing stages. While extended encoding supports resistance to early distraction, maintenance-stage distractions exert persistent effects—especially when distractors are similar to targets. Our results highlight the importance of presentation duration and distractor similarity in shaping VWM robustness, and suggest that distinct cognitive mechanisms may underlie suppression at encoding and maintenance stages.

**Supplementary Information:**

The online version contains supplementary material available at 10.1038/s41598-025-18699-z.

## Introduction

Visual working memory (VWM) is essential for everyday cognitive processes, as it allows us to temporarily hold and manipulate visual information. This system is crucial for activities like reading, navigation, decision-making, and even social interactions, as it helps us remember and process visual information about our environment^1^. One of the critical aspects of VWM is its limited capacity—typically only 3–4 items can be stored simultaneously^2–4^. Although strategies such as grouping and attention resource allocation can improve VWM performance to some extent^5––12^, the fundamental capacity limit appears unavoidable. This limited storage space means that the brain must prioritize important information and ignore irrelevant details to maximize efficiency^13^. This is where the mechanism of distractor filtering in VWM becomes essential. Filtering out distractions allows the brain to focus only on relevant stimuli, enhancing VWM’s efficiency and enabling us to concentrate on the visual information that is most pertinent to the task at hand. Thus, there is a growing body of research investigating how individuals process distractor information and its influence on VWM mechanisms^14–17^.

In traditional research of VWM, a typical experimental paradigm involves first presenting participants with a memory array containing several visual stimuli, which they are instructed to remember. After the stimuli disappear, there is a blank interval delay during which participants are required to maintain the memory targets in VWM. Following this delay, a test array appears on the screen, and participants respond based on the information held in VWM^18^. Previous research on distractor filtering within VWM can be categorized according to the stage at which distractor stimuli appear: some studies present distractors concurrently with the memory array (encoding-stage distraction)^13,19–22^, while others introduce distractors only after the memory array has disappeared, during the delay interval (delay-stage distraction)^16,23^. In the encoding-stage distraction paradigm, distractor processing occurs simultaneously with the encoding of memory targets. We refer to this condition as “encoding-distraction.” Conversely, in delay-stage distraction studies, distractor processing occurs after the stimuli have disappeared and during the blank interval delay, which we term this condition “delay-distraction.”

Research on encoding-distraction often uses event-related potentials (ERPs) to investigate how participants manage distractors during VWM encoding. The contralateral delay activity (CDA) ERP component^24–32^, which reflects the VWM load, has been widely used to examine the relationship between distraction resistance and VWM capacity. Vogel, et al.^13^, for instance, demonstrated that individuals with lower VWM capacity tend to encode simple distractors (e.g., color or orientation), while those with higher capacity more effectively ignore these distractors, suggesting a link between VWM capacity and distractor resistance during encoding. Thus, the degree of disruption from encoding-stage distractors appears to correlate with individual VWM capacity.

Substantial evidence has also emerged from studies focusing on delay-distraction. For instance, Hakim, et al.^23^ conducted a change detection task in which participants memorized six simple stimuli, with distractors presented during the delay period—after the memory array and before the test array. Their findings showed reduced task performance under delay-stage distraction, emphasizing that distractions during this stage can significantly impair VWM performance.

However, the effects of encoding- versus delay-stage distractors on VWM may differ markedly. Duan, et al.^33^, for example, conducted a systematic investigation examining individual resilience against distractors at both stages. Using a continuous recall task, they assessed the effects of distractors presented during encoding versus delay on the recall of simple stimuli (e.g., teardrop orientations). Their findings indicated that VWM performance was significantly impaired only by delay-stage distractors, with encoding-stage distractions not adversely impacting performance. In our recent study^34^, we extended this by using facial stimuli in a change detection task, presenting neutral face distractors either during encoding or delay stages, to examine the impact of complex distractors at each stage on VWM processing. Results similarly showed significant impairment from delay-stage distractors but not encoding-stage distractors. Thus, these findings suggest that stage-specific mechanisms underlie distractor influence on VWM maintenance, with delay-stage distractors exerting a significant impact on performance.

Additionally, our previous research suggests that the length of stimulus presentation influences the representational state of VWM^35^. By manipulating presentation duration of memory stimuli, it is possible to place VWM consolidation at different temporal stages. VWM consolidation can be broadly divided into early and late stages, each relying on distinct mechanisms for allocating memory resources to VWM representations^36–39^. In a recent ERP study on encoding-stage distraction^22^, we investigated distractor suppression by analyzing the distractor-induced ERP component (PD)^40,41^. By manipulating the presentation duration of target and distractor stimuli, we examined differences in how participants process encoding-distractors across different VWM consolidation stages. Results indicated that with sufficient time to consolidate target stimuli, participants could more effectively suppress distractors, suggesting that distractor filtering may depend on the presentation duration, at least in encoding-distraction contexts.

Notably, previous encoding-distraction studies typically used brief stimulus presentations (e.g., 100–200 ms)^13,20,42^. In contrast, both Duan, et al.^33^ and Ye, et al.^34^’s studies used longer presentation duration (1000 ms) for target and distractor stimuli. This experimental setup variation may account for conflicting findings in prior encoding-distraction research, where some evidence suggests that encoding-distractors impair VWM performance^20^— a result not replicated in recent studies^33,34^. However, no research has yet directly manipulated presentation duration to examine its effect on encoding- and delay-distraction processing within VWM.

This study aims to examine how the presentation duration of stimuli affects the impact of distractors presented at different stages of VWM processing. We manipulate the presentation duration for memory and distractor stimuli and investigate how variations in duration of memory targets and distractors influence VWM performance when distractions occur during the encoding versus the delay stage. This approach enables us to explore whether the length of stimulus consolidation affects the mechanisms by which individuals filter distractors at different VWM processing stages. We hypothesize two potential outcomes. First, if presentation duration indeed modulates the distractor filtering mechanism, then the extent of VWM performance impairment caused by distractors should vary according to duration. Specifically, we expect delay-stage distractors to substantially impair VWM performance regardless of presentation duration. In contrast, encoding-stage distractors should only impair performance at shorter duration, where consolidation is insufficient. With longer duration allowing for full consolidation, encoding-stage distractors are less likely to impact VWM performance. Alternatively, if presentation duration does not significantly modulate distractor filtering, the qualitative difference between encoding- and delay-stage distractors on VWM performance should remain consistent across presentation duration.

Additionally, the studies by Duan, et al.^33^ and Ye, et al.^34^ varied not only in visual stimuli but also in task type. Duan, et al.^33^ used a continuous recall task requiring participants to memorize the orientations of three targets and then recall one target’s orientation angle accurately at test, thus demanding high memory precision for each item. In contrast, Ye, et al.^34^ used a change detection task that required participants to determine if the test array exactly matched the memory array, allowing for successful task completion even with lower memory precision for each item. Although both studies found consistent results, each used a longer presentation duration (1000 ms), leaving it unclear whether the same stage-specific distractor effects would hold under shorter presentation duration across tasks with different precision demands. Therefore, in Experiment 1, participants complete both a continuous recall task and a change detection task to examine how memory precision requirements interact with presentation duration to influence distractor effects across different VWM stages.

## Experiment 1

To examine whether stimulus presentation duration modulates individual processing of encoding-distraction and delay-distraction, participants completed a continuous recall task and a change detection task. In both tasks, participants were instructed to memorize three target orientations while ignoring the potential presence of three orientation distractors. We manipulated the factors of distractor presentation condition and presentation duration of stimuli. For the distractor presentation condition, four different distractor conditions were included: a no-distraction condition, an encoding-distraction condition, a full-distraction condition, and a delay-distraction condition. In the no-distraction condition, no distractors appeared. In the encoding-distraction condition, three distractors were presented alongside the targets in the memory array and disappeared simultaneously with the targets at the end of the encoding stage. In the full-distraction condition, three distractors appeared with the targets during the memory array presentation; however, unlike the encoding-distraction condition, the distractors persisted after the encoding stage until the test array appeared. In the delay-distraction condition, no distractors were present during the memory array, but three distractors appeared during the delay stage after the memory array disappeared. This experimental setup allowed us to compare VWM performance under different distraction conditions (during either the encoding stage, the delay stage, or both stages) against a no-distraction baseline. If the presence of distractors at a specific stage induced a significant distraction effect, we expected VWM performance in that condition to be significantly worse than in the no-distraction condition. Additionally, for the presetnation duration manipulation, we selected a short presentation duration of 200 ms, consistent with previous encoding-distraction research^20^, and a long presentation duration of 1000 ms, as used in the studies by Duan, et al.^33^ and Ye, et al.^34^. It is worth noting that in these previous studies, the duration of distractor presentation was typically matched to the duration of the memory array, regardless of whether distraction occurred during encoding or delay stage. For instance, when Duan, et al.^33^ and Ye, et al.^34^ presented distractors only during the delay period, the distractors remained on-screen for the same 1000 ms duration as the memory array. To maintain consistency with this approach, we also adjusted the delay-stage distractor duration to match the memory array duration in each condition. Specifically, in the short presentation condition, delay distractors were shown for 200 ms; in the long presentation condition, they were presented for 1000 ms. This ensured that the durations of distraction during encoding and delay were equivalent within each level of presentation duration.

### Methods

#### Participants

To ensure sufficient statistical power for the t-test comparisons, we conducted an a priori power analysis using G*Power 3.1.9.2. This analysis was informed by the expected effect size based on the study by Duan, et al.^33^. Assuming a large effect size (Cohen’s d = 0.80) for our design, with a power of 80% and an alpha level of 0.05, the analysis indicated a minimum required sample size of 15 participants.

Our study adhered to the principles of the Declaration of Helsinki and received ethics approval from the Ethics Committee of Sichuan Normal University. Thirty-one college students participated in the study in exchange for compensation. However, one participant was excluded due to a program crash during the task, and two additional participants were removed due to accuracy in the change detection task below chance level (0.5), resulting in a final sample of 28 valid participants (2 males and 26 females; mean age = 20.29 years, SD = 1.212, age range 19–23 years) included in the data analyses. This sample size closely aligns with that used in the studies by Duan, et al.^33^ (*N* = 24) and Ye, et al.^34^ (*N* = 26). All participants reported normal or corrected-to-normal vision, normal color vision, and no history of neurological conditions. Written informed consent was obtained from each participant prior to the study.

### Materials

We used arrows as stimuli (1.0° × 0.5° visual angle) for both targets and distractors. Each arrow’s orientation was randomly selected between 0° and 359°, with at least a 30° orientation difference between any two arrows to prevent overlap or similar orientations. Targets and distractors were distinguished by color (red [RGB: 255, 0, 0] or blue [RGB: 0, 0, 255]). Stimuli appeared on a gray (RGB: 128, 128, 128) background, with arrows distributed within an invisible rectangle (4.0° × 6.0°), ensuring a minimum of 1.6° spacing between any two arrows. The experiment was programmed using E-Prime software (E-prime 2.0, Psychology Software Tools, Inc.), and participants were seated 70 cm from a 17-inch screen in a dark, soundproof room.

### Procedure

To examine the effect of target encoding time on distraction processing, we manipulated two factors: target presentation duration (short and long) and the type of distraction (no-distraction, encoding-distraction, full-distraction, and delay-distraction). Each participant completed both a continuous recall task and a change detection task, always performing the continuous recall task first.

**Fig. 1:**
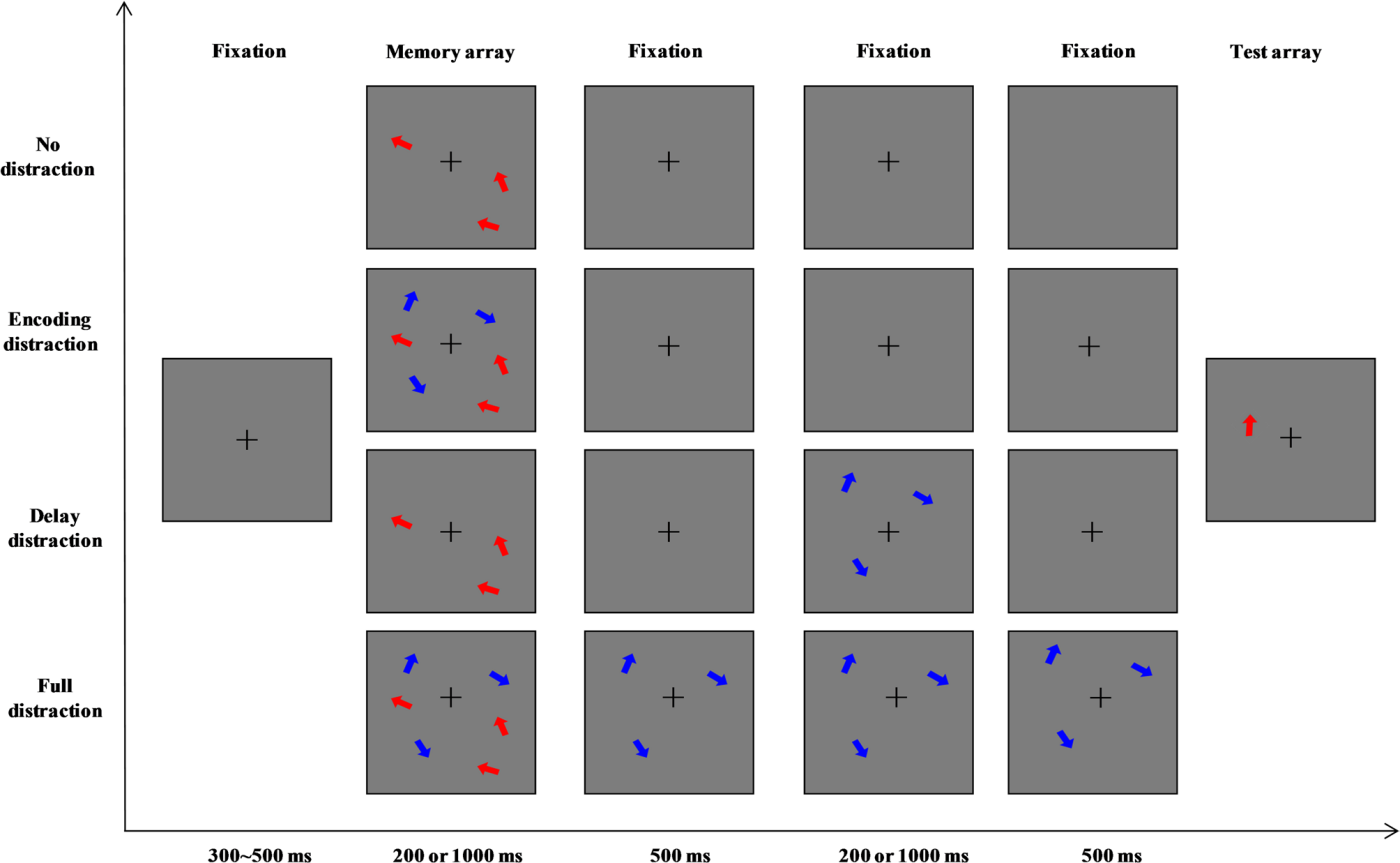
Schematic trial structure of the VWM task used in Experiment 1. Four distraction conditions (no distraction, encoding distraction, delay distraction, and full distraction) under short (200 ms) and long (1000 ms) presentation durations were included in both continuous recall task and change detection task. Each trial began with a fixation display, followed by a memory array consisting of three red arrows (targets), which was either for a short duration (200 ms) or for a long duration (1000 ms). Depending on the condition, distractors (blue arrows) were presented either concurrently with the memory array (encoding distraction), during the delay period (delay distraction), or throughout both periods (full distraction). No distractors were shown in the baseline (no distraction) condition. After a delay interval (1200 ms for short and 2000 ms for long presentation), a single probe arrow was displayed. This structure was shared by both the continuous recall and change detection tasks, differing only in the test display. In the continuous recall task, participants adjusted the orientation of the probe arrow to match the remembered target (the probe remained on screen until response). In the change detection task, participants judged whether the probe arrow matched the orientation of the original target at the same location (the probe remained on screen until response or 2500 ms).

The trial structure of Experiment 1 is shown in Fig. 1. In both tasks, participants memorized three target arrows in a memory array while ignoring distractor arrows. Target and distractor colors (red or blue) were assigned and counterbalanced across participants. In the baseline condition (no-distraction), each trial began with a fixation cross (1.5° × 1.5°) presented for 300–500 ms, followed by three arrows with varying orientations displayed for either 200 ms or 1000 ms. Participants were asked to remember these orientations. After a blank delay (1200 ms blank for the short presentation duration condition or 2000 ms blank for the long presentation duration condition), a test array was presented. In the continuous recall task, the test array contained a single arrow pointing vertically upward (always presented at 0° orientation) at one of the original target locations. Participants were instructed to adjust the arrow’s orientation using the computer mouse to match the orientation of the corresponding target from the memory array. After each trial, participants received feedback on the orientation offset (difference from the target). The next trial began 400–600 ms after participants acknowledged the feedback. In the change detection task, this test array contained one arrow at one of the original target positions. In half the trials, the test arrow’s orientation differed by 30–60° from the target’s, while in the remaining trials, it matched the target exactly. Participants indicated whether the test arrow’s orientation matched the target’s. The next trial began 100 ms after participants responsed.

In the encoding-distraction condition, the procedure was identical to the no-distraction condition, except that, during the memory array presentation, three distractor arrows appeared alongside the targets in a different color. Participants were instructed to remember only the target arrows (red for half the participants, blue for the other half). In the short presentation condition, distractor arrows appeared for 200 ms alongside the target arrows, while in the long presentation condition, distractors appeared for 1000 ms and disappeared at the same time as the targets.

In the full-distraction condition, the setup was similar to the encoding-distraction condition, except that the distractors remained visible during the delay period after the targets disappeared, up until the test array presentation. For the short presentation condition, distractors remained for an additional 1200 ms after the memory array; for the long presentation condition, they remained for an additional 2000 ms.

In the delay-distraction condition, the setup was similar to the no-distraction condition except during the delay period. Here, after the memory array disappeared, a 500 ms blank interval was followed by three distractor arrows appearing in new positions for 200 ms (short presentation condition) or 1000 ms (long presentation condition). A second 500 ms blank interval then preceded the test array.

Both tasks used a within-subject design with factors for presentation duration (short and long) and distraction condition (no-distraction, encoding-distraction, full-distraction, delay-distraction). The continuous recall task included 100 trials per condition, totaling 800 trials, while the change detection task comprised 48 trials per condition, totaling 384 trials. Participants took a 5-minute break between the two tasks. The entire experiment lasted approximately 90 min, and each task included 18 practice trials to ensure participants understood the procedure.

#### Data analysis

We analyzed the results separately for the continuous recall task and the change detection task. For the continuous recall task, memory performance was indexed by the absolute angular error between the reported and actual orientation of the target, referred to as the offset. Larger offsets indicated poorer memory performance. For the change detection task, the primary dependent measure was response accuracy (ACC) for each condition.

For both tasks, we conducted a two-way repeated-measures ANOVA with presentation duration (short vs. long) and distraction condition (no-distraction, encoding-distraction, full-distraction, delay-distraction) as within-subject factors.

To assess the effects of distraction, we performed planned pairwise t-tests comparing each distraction condition with the no-distraction baseline. Additionally, we compared performance between short and long duration within each distraction condition to examine how encoding time modulated distraction effects.

We also calculated distraction costs by computing the performance difference between each distraction condition and the no-distraction baseline. Specifically:

For the continuous recall task (offset):

$$\:{\text{O}\text{f}\text{f}\text{s}\text{e}\text{t}}_{\text{E}\text{n}\text{c}\text{o}\text{d}\text{i}\text{n}\text{g-}\text{d}\text{i}\text{s}\text{t}\text{r}\text{a}\text{c}\text{t}\text{i}\text{o}\text{n}\:\text{c}\text{o}\text{s}\text{t}}={\text{O}\text{f}\text{f}\text{s}\text{e}\text{t}}_{\text{E}\text{n}\text{c}\text{o}\text{d}\text{i}\text{n}\text{g-}\text{d}\text{i}\text{s}\text{t}\text{r}\text{a}\text{c}\text{t}\text{i}\text{o}\text{n}} - \:{\text{O}\text{f}\text{f}\text{s}\text{e}\text{t}}_{\text{N}\text{o-}\text{d}\text{i}\text{s}\text{t}\text{r}\text{a}\text{c}\text{t}\text{i}\text{o}\text{n}}$$

$$\:{\text{O}\text{f}\text{f}\text{s}\text{e}\text{t}}_{\text{F}\text{u}\text{l}\text{l-}\text{d}\text{i}\text{s}\text{t}\text{r}\text{a}\text{c}\text{t}\text{i}\text{o}\text{n}\:\text{c}\text{o}\text{s}\text{t}}={\text{O}\text{f}\text{f}\text{s}\text{e}\text{t}}_{\text{F}\text{u}\text{l}\text{l-}\text{d}\text{i}\text{s}\text{t}\text{r}\text{a}\text{c}\text{t}\text{i}\text{o}\text{n}}-\:{\text{O}\text{f}\text{f}\text{s}\text{e}\text{t}}_{\text{N}\text{o-}\text{d}\text{i}\text{s}\text{t}\text{r}\text{a}\text{c}\text{t}\text{i}\text{o}\text{n}}$$

$$\:{\text{O}\text{f}\text{f}\text{s}\text{e}\text{t}}_{\text{D}\text{e}\text{l}\text{a}\text{y-}\text{d}\text{i}\text{s}\text{t}\text{r}\text{a}\text{c}\text{t}\text{i}\text{o}\text{n}\:\text{c}\text{o}\text{s}\text{t}}={\text{O}\text{f}\text{f}\text{s}\text{e}\text{t}}_{\text{D}\text{e}\text{l}\text{a}\text{y-}\text{d}\text{i}\text{s}\text{t}\text{r}\text{a}\text{c}\text{t}\text{i}\text{o}\text{n}}-\:{\text{O}\text{f}\text{f}\text{s}\text{e}\text{t}}_{\text{N}\text{o-}\text{d}\text{i}\text{s}\text{t}\text{r}\text{a}\text{c}\text{t}\text{i}\text{o}\text{n}}$$

For the change detection task (ACC):

$$\:{\text{A}\text{C}\text{C}}_{\text{E}\text{n}\text{c}\text{o}\text{d}\text{i}\text{n}\text{g-}\text{d}\text{i}\text{s}\text{t}\text{r}\text{a}\text{c}\text{t}\text{i}\text{o}\text{n}\:\text{c}\text{o}\text{s}\text{t}}={\text{A}\text{C}\text{C}}_{\text{N}\text{o-}\text{d}\text{i}\text{s}\text{t}\text{r}\text{a}\text{c}\text{t}\text{i}\text{o}\text{n}}-\:{\text{A}\text{C}\text{C}}_{\text{E}\text{n}\text{c}\text{o}\text{d}\text{i}\text{n}\text{g-}\text{d}\text{i}\text{s}\text{t}\text{r}\text{a}\text{c}\text{t}\text{i}\text{o}\text{n}}$$

$$\:{\text{A}\text{C}\text{C}}_{\text{F}\text{u}\text{l}\text{l-}\text{d}\text{i}\text{s}\text{t}\text{r}\text{a}\text{c}\text{t}\text{i}\text{o}\text{n}\:\text{c}\text{o}\text{s}\text{t}}={\text{A}\text{C}\text{C}}_{\text{N}\text{o-}\text{d}\text{i}\text{s}\text{t}\text{r}\text{a}\text{c}\text{t}\text{i}\text{o}\text{n}}-\:{\text{A}\text{C}\text{C}}_{\text{F}\text{u}\text{l}\text{l-}\text{d}\text{i}\text{s}\text{t}\text{r}\text{a}\text{c}\text{t}\text{i}\text{o}\text{n}}$$

$$\:{\text{A}\text{C}\text{C}}_{\text{D}\text{e}\text{l}\text{a}\text{y-}\text{d}\text{i}\text{s}\text{t}\text{r}\text{a}\text{c}\text{t}\text{i}\text{o}\text{n}\:\text{c}\text{o}\text{s}\text{t}}={\text{A}\text{C}\text{C}}_{\text{N}\text{o-}\text{d}\text{i}\text{s}\text{t}\text{r}\text{a}\text{c}\text{t}\text{i}\text{o}\text{n}}-\:{\text{A}\text{C}\text{C}}_{\text{D}\text{e}\text{l}\text{a}\text{y-}\text{d}\text{i}\text{s}\text{t}\text{r}\text{a}\text{c}\text{t}\text{i}\text{o}\text{n}}$$

A positive distraction cost—whether in offset or accuracy—indicates performance impairment caused by the distractor, with larger values reflecting greater disruption.

To further explore the pattern of distraction costs, we performed an additional two-way repeated-measures ANOVA with presentation duration (short vs. long) and cost type (encoding, full, delay) as within-subject factors. Planned comparisons were also conducted to examine differences in distraction cost across conditions and presentation duration.

Effect sizes were reported as partial eta squared (η^2^_p_) for ANOVAs and Cohen’s d for t-tests. In addition, we conducted Bayes factor analyses to quantify the strength of evidence for the alternative versus the null hypothesis^43^. The Bayes factor (BF_10_) provides an odds ratio for the likelihood of the alternative versus the null hypothesis, where values < 1 favor the null hypothesis, and values > 1 favor the alternative hypothesis. For example, a BF_10_ of 0.25 would suggest the null hypothesis is four times more likely than the alternative.

## Results

### Continuous recall task

#### Offset

**Fig. 2:**
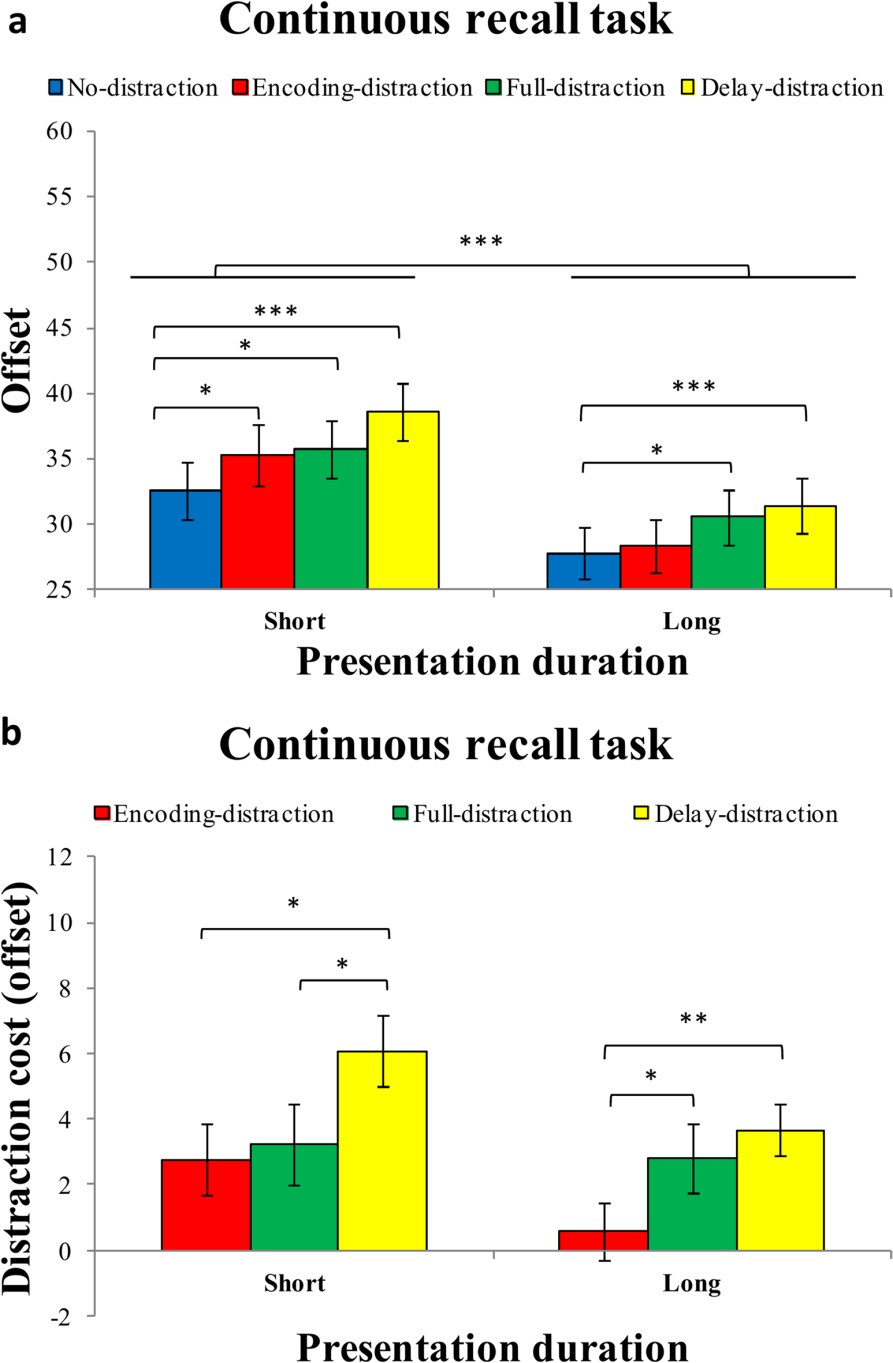
Results of the continuous recall task in Experiment 1. (**a**) Mean recall error (offset in degrees) as a function of presentation duration (short vs. long) and distraction condition (no distraction, encoding distraction, full distraction, delay distraction) in the continuous recall task of Experiment 1. (**b**) Distraction cost (i.e., offset relative to the no-distraction condition) under each distraction condition (encoding distraction, full distraction, delay distraction) for both short and long presentation durations. Error bars represent ± 1 standard error of the mean (SEM). *p < 0.05; **p < 0.01; ***p < 0.001.

The mean offset for each distraction condition (no-distraction vs. encoding-distraction vs. full-distraction vs. delay-distraction) under short or long presentation duration is presented in Fig. 2a. The ANOVA on offset revealed a significant main effect of presentation duration, F (1,27) = 51.659, *p* < 0.001, η^2^_p_ = 0.657, and a significant main effect of the distraction condition, F (3,81) = 12.754, *p* < 0.001, η^2^_p_ = 0.321. However, no significant interaction on offset was found between the presentation duration and distraction condition, F (3,81) = 1.520, *p* = 0.220, η^2^_p_ = 0.053.

Planned pairwise comparisons revealed that, under the short presentation duration, the offset in the no-distraction condition was significantly lower than in the encoding-distraction condition, t(27) = 2.529, *p* = 0.018, Cohen’s d = 0.478, BF_10_ = 2.871; the full-distraction condition, t(27) = 2.644, *p* = 0.013, Cohen’s d = 0.500, BF_10_ = 13.587; and the delay-distraction condition, t(27) = 5.719, *p* < 0.001, Cohen’s d = 1.081, BF_10_ > 1000. These results indicate that all forms of distraction, regardless of when they occurred, impaired VWM performance when encoding time was limited—with delay-stage distractors causing the greatest disruption. However, under the long presentation duration, no significant difference in offset was observed between the no-distraction and encoding-distraction conditions, t(27) = 0.650, *p* = 0.521, Cohen’s d = 0.123, BF_10_ = 0.243. In contrast, the offset in the no-distraction condition was significantly lower than in the full-distraction condition, t(27) = 2.648, *p* = 0.013, Cohen’s d = 0.500, BF_10_ = 3.610, and the delay-distraction condition, t(27) = 4.730, *p* < 0.001, Cohen’s d = 0.894, BF_10_ = 399.497. This pattern suggests that when encoding time was sufficient, participants were able to resist encoding-stage distraction, but full and especially delay-stage distractions continued to impair VWM performance.

Additionally, the offset for the long presentation duration was significantly lower than that for the short presentation duration across all conditions: no-distraction, t(27) = 3.861, *p* < 0.001, Cohen’s d = 0.730, BF_10_ = 50.12; encoding-distraction, t(27) = 5.448, *p* < 0.001, Cohen’s d = 1.029, BF_10_ > 1000; full-distraction, t(27) = 4.436, *p* < 0.001, Cohen’s d = 0.838, BF_10_ = 196.34; and delay-distraction, t(27) = 6.624, *p* < 0.001, Cohen’s d = 1.252, BF_10_ > 1000. These results confirm that longer encoding time generally enhances memory performance.

#### Distraction cost (offset)

The mean distraction cost (offset) for each distraction condition (encoding-distraction, full-distraction, and delay-distraction) under short and long presentation durations is shown in Fig. 2b. A two-way repeated-measures ANOVA on distraction cost revealed a significant main effect of distraction type, F (2,54) = 20.168, *p* < 0.001, η^2^_p_ = 0.428. The distraction cost was significantly smaller in the encoding-distraction condition (M = 1.677, SD = 3.697) compared to the delay-distraction condition (M = 4.884, SD = 3.823), t(27) = 3.554, *p* < 0.001, Cohen’s d = 0.672, BF_10_ = 24.794. Similarly, the distraction cost in the full-distraction condition (M = 3.008, SD = 4.437) was also significantly smaller than in the delay-distraction condition, t(27) = 2.048, *p* = 0.05, Cohen’s d = 0.387, BF_10_ = 1.221. However, no significant difference was found between the encoding- and full-distraction conditions, t(27) = 1.644, *p* = 0.112, Cohen’s d = 0.311, BF_10_ = 0.661. There was no main effect of presentation duration, F (1,27) = 0.236, *p* = 0.631, η^2^_p_ = 0.009, nor a significant interaction between presentation duration and distraction type, F (2,54) = 1.617, *p* = 0.208, η^2^_p_ = 0.057.

Planned pairwise comparisons revealed that under short presentation duration, participants showed significantly larger distraction costs in the delay-distraction condition compared to the encoding-distraction condition, t(27) = 2.54, *p* = 0.017, Cohen’s d = 0.480, BF_10_ = 2.927 and the full-distraction condition, t(27) = 2.41, *p* = 0.023, Cohen’s d = 0.455, BF_10_ = 2.292. The encoding- and full-distraction conditions did not significantly differ from each other, t(27) = 0.37, *p* = 0.713, Cohen’s d = 0.070, BF_10_ = 0.214.

Under long presentation duration, a different pattern emerged. Distraction cost in the encoding-distraction condition was significantly smaller than in both the full-distraction condition, t(27) = 2.73, *p* = 0.011, Cohen’s d = 0.516, BF_10_ = 4.233 and the delay-distraction condition, t(27) = 2.97, *p* = 0.006, Cohen’s d = 0.561, BF_10_ = 6.902. However, no significant difference was observed between the full- and delay-distraction conditions, t(27) = 0.81, *p* = 0.424, Cohen’s d = 0.153, BF_10_ = 0.271. This result suggests that sufficient encoding time enabled participants to better resist early distractors but did not fully protect against interference from later ones.

In addition, we compared distraction costs across presentation durations within each condition. None of these comparisons reached significance: encoding-distraction, t(27) = 1.53, *p* = 0.138, Cohen’s d = 0.289, BF_10_ = 0.566; full-distraction, t(27) = 0.28, *p* = 0.782, Cohen’s d = 0.053, BF_10_ = 0.208; delay-distraction, t(27) = 2.04, *p* = 0.052, Cohen’s d = 0.385, BF_10_ = 1.198. These results overall suggest that presentation duration had limited influence on the size of distraction effects.

### Change detection task

#### Accuracy

**Fig. 3:**
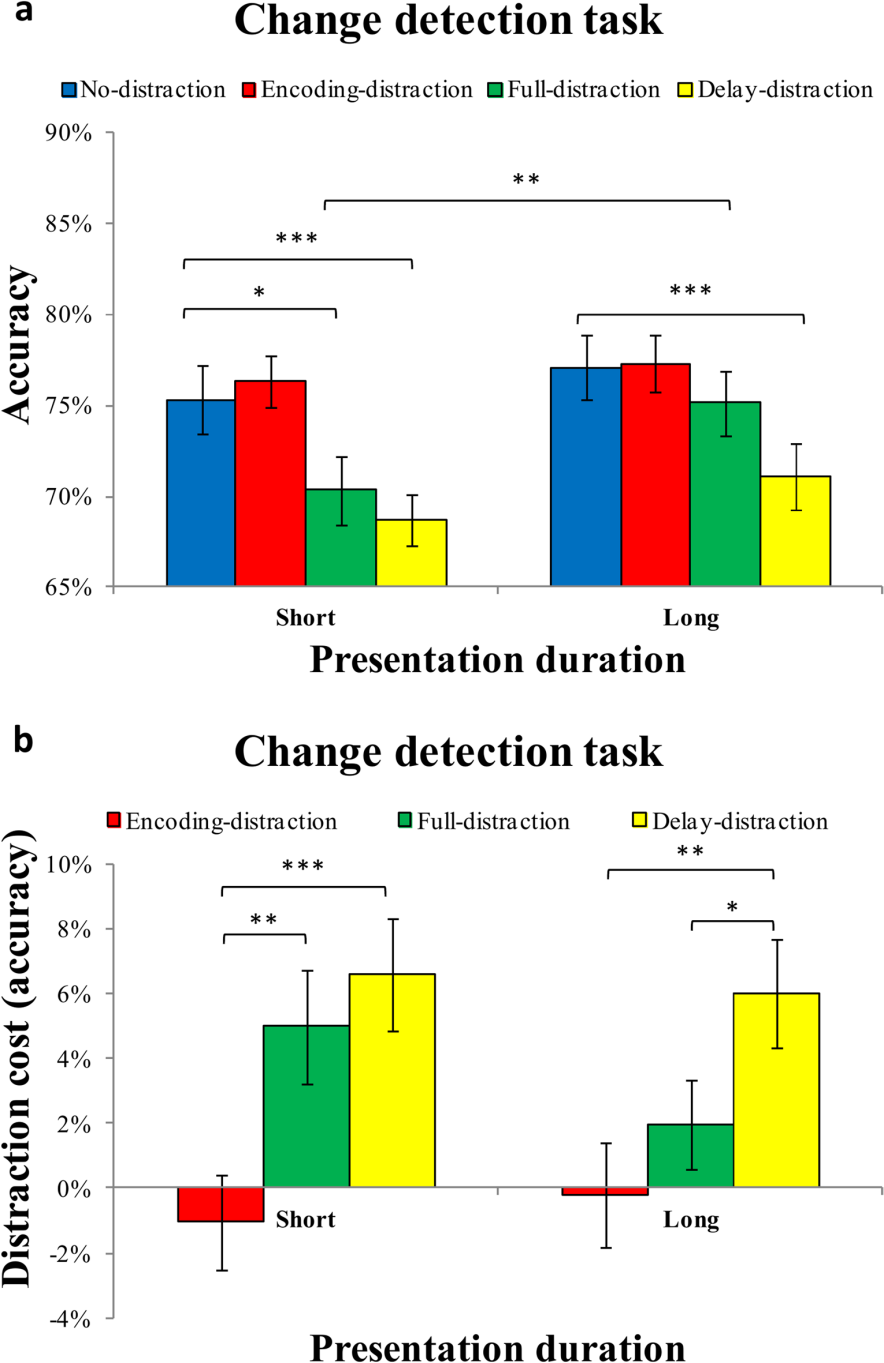
Results of the change detection task in Experiment 1. (**a**) Mean accuracy in the change detection task as a function of presentation duration (short vs. long) and distraction condition (no distraction, encoding distraction, full distraction, delay distraction) in Experiment 1. (**b**) Distraction cost (i.e., accuracy difference from the no-distraction condition) across distraction conditions (encoding distraction, full distraction, delay distraction) for each presentation duration. Error bars represent ± 1 standard error of the mean (SEM). *p < 0.05; **p < 0.01; ***p < 0.001.

The mean accuracy for each distraction condition (no-distraction vs. encoding-distraction vs. full-distraction vs. delay-distraction) under short or long presentation duration is presented in Fig. 3a. The ANOVA revealed a significant main effect of presentation duration, F (1,27) = 9.479, *p* = 0.005, η^2^_p_ = 0.260, and a significant main effect of the distraction condition, F (3,81) = 16.969, *p* < 0.001, η^2^_p_ = 0.386. However, no significant interaction was found between the presentation duration and distraction condition, F (3,81) = 1.101, *p* = 0.352, η^2^_p_ = 0.039.

Planned pairwise comparisons indicated that, under the short presentation duration, accuracy in the no-distraction condition was significantly higher than in the full-distraction condition, t(27) = 2.830, *p* = 0.009, Cohen’s d = 0.535, BF_10_ = 5.194, and the delay-distraction condition, t(27) = 3.845, *p* < 0.001, Cohen’s d = 0.727, BF_10_ = 48.281. However, no significant difference in accuracy was observed between the no-distraction and encoding-distraction conditions, t(27) = 0.707, *p* = 0.486, Cohen’s d = 0.134, BF_10_ = 0.252. This suggests that brief encoding-stage distractors did not impair performance, whereas later or sustained distractors did reduce accuracy under limited exposure. Under the long presentation duration, accuracy in the no-distraction condition remained significantly higher than in the delay-distraction condition, t(27) = 43.570, *p* < 0.001, Cohen’s d = 0.675, BF_10_ = 25.659, but showed no significant differences with the encoding-distraction condition, t(27) = 0.134, *p* = 0.894, Cohen’s d = 0.025, BF_10_ = 0.202, or the full-distraction condition, t(27) = 1.430, *p* = 0.164, Cohen’s d = 0.270, BF_10_ = 0.499. This pattern indicates that with sufficient encoding time, participants were largely resilient to distraction occurring during or immediately following encoding, but still vulnerable to distraction during the delay period.

Additionally, accuracy for the long presentation duration was significantly higher than that for the short presentation duration only in the full-distraction condition, t(27) = 3.491, *p* = 0.002, Cohen’s d = 0.660, BF_10_ = 21.486. In contrast, no significant differences in accuracy were found between long and short presentation durations in the no-distraction condition, t(27) = 1.043, *p* = 0.306, Cohen’s d = 0.197, BF_10_ = 0.328; encoding-distraction condition, t(27) = 0.699, *p* = 0.491, Cohen’s d = 0.132, BF_10_ = 0.251; or delay-distraction condition, t(27) = 1.300, *p* = 0.205, Cohen’s d = 0.246, BF_10_ = 0.428.

#### Distraction cost (ACC)

The mean distraction cost (ACC) for each distraction condition (encoding-distraction, full-distraction, and delay-distraction) under short and long presentation durations is shown in Fig. 3b. The ANOVA on distraction cost (ACC) revealed a significant main effect of the distraction condition, F (2,54) = 6.747, *p* = 0.003, η^2^_p_ = 0.200. The distraction cost in the encoding-distraction condition (M = -0.006, SD = 0.060) was significantly lower than that in the full-distraction condition (0.03482 ± 0.05753), t(27) = 3.811, *p* < 0.001, Cohen’s d = 0.72, BF_10_ = 44.62, and also significantly lower than that in the delay-distraction condition (M = 0.063, SD = 0.060), t(27) = 6.766, *p* < 0.001, Cohen’s d = 1.279, BF_10_ > 1000. Moreover, the distraction cost in the full-distraction condition was significantly lower than in the delay-distraction condition, t(27) = 2.384, *p* = 0.024, Cohen’s d = 0.45, BF_10_ = 2.189. However, no significant main effect of the presentation duration, F (1,27) = 2.170, *p* = 0.152, η^2^_p_ = 0.074., and no significant interation was found between the presentation duration and distraction condition, F (2,54) = 1.198, *p* = 0.309, η^2^_p_ = 0.042.

To better understand these effects, planned comparisons were conducted separately for each presentation duration condition. Under short presentation duration, the distraction cost in the encoding-distraction condition was significantly lower than in the full-distraction condition, t(27) = 3.85, *p* = 0.001, Cohen’s d = 0.727, BF_10_ = 48.539, and was also significantly lower than in the delay-distraction condition, t(27) = 6.22, *p* < 0.001, Cohen’s d = 1.176, BF_10_ > 1000. However, the difference between the full- and delay-distraction conditions did not reach significance, t(27) = 0.99, *p* = 0.331, Cohen’s d = 0.187, BF_10_ = 0.313, suggesting that delay-stage distractors were not reliably more disruptive than full-interval distractors when encoding time was limited.

Under long presentation duration, the distraction cost in the delay-distraction condition remained significantly greater than in the encoding-distraction condition, t(27) = 3.62, *p* = 0.001, Cohen’s d = 0.684, BF_10_ = 28.626, and also exceeded that of the full-distraction condition, t(27) = 2.46, *p* = 0.020, Cohen’s d = 0.466, BF_10_ = 2.537. In contrast, the encoding- and full-distraction conditions did not significantly differ, t(27) = 1.53, *p* = 0.138, Cohen’s d = 0.289, BF_10_ = 0.566, indicating that when stimulus presentation was extended, full-interval distraction no longer produced reliably more impairment than encoding-only distraction.

In addition, comparisons across presentation durations revealed no significant differences in distraction cost between short and long exposure durations for any of the three conditions: encoding-distraction, t(27) = 0.40, *p* = 0.694, Cohen’s d = 0.075, BF_10_ = 0.216; full-distraction, t(27) = 1.32, *p* = 0.198, Cohen’s d = 0.249, BF_10_ = 0.438; and delay-distraction, t(27) = 0.24, *p* = 0.812, Cohen’s d = 0.045, BF_10_ = 0.206. This suggests that prolonging stimulus exposure did not systematically reduce distraction-related performance costs in any specific condition.

### Discussion

In Experiment 1, participants performed both a continuous recall task and a change detection task. The continuous recall task required participants to recall the orientation of specified targets with high precision, making it a task that demands high visual VWM precision for optimal performance. In contrast, the change detection task only required participants to have a low-precision memory of target items to determine if a change had occurred, resulting in lower VWM precision demands. Accordingly, the results of Experiment 1 allowed us to observe whether different stages of distractor presentation impaired VWM performance under conditions requiring high versus low memory precision.

In the continuous recall task, the offset results indicate that any form of distraction, regardless of when it occurred, impaired VWM performance under the short presentation duration condition. This suggests that when VWM precision demands are high and encoding time is limited, both encoding-stage and delay-stage distractors disrupt memory performance. The presence of encoding-distraction effects aligns with previous findings showing that distractions during encoding can impair VWM performance^20^. Moreover, we observed that delay-stage distraction caused significantly greater impairment than both encoding- and full-distraction conditions. However, with longer stimulus presentation duration, only delay-distraction effects were evident, while encoding- stage distraction effects were not observed, consistent with previous results from the study by Duan, et al.^33^ which used a continuous recall task. Additionally, both full- and delay-distraction conditions led to significantly greater impairment than the encoding-distraction condition. Taken together, these results suggest that in tasks with high precision demands, delay-stage distractors consistently impair VWM and produce the greatest performance cost, while the effect of encoding-stage distraction depends on presentation duration—emerging only under shorter exposure durations.

In contrast, the accuracy results for the change detection task suggest that during short presentation duration, encoding-only distractions did not impair VWM, whereas both full- and delay-distractions did. With longer exposures, only delay distractions continued to impair VWM performance. This indicates that in tasks with lower memory precision demands, delay-stage distractors consistently impair VWM performance. During shorter stimulus presentations, individuals can only effectively resist the impact of encoding-stage distractions, while during longer presentations, they can resist both encoding-stage and continuously present distractions. These findings are consistent with those reported by Ye, et al.^34^, which also used a change detection task. Across both presentation duration conditions, delay-stage distraction caused the most pronounced impairment in memory performance, mirroring the pattern observed in the continuous recall task.

The differing result patterns between the continuous recall and change detection tasks highlight that task demands on memory precision influence an individual’s ability to resist distractor effects. For high-precision memory tasks, filtering distractions may be more challenging. When high memory precision is required, distractions presented during shorter stimulus presentation duration in the encoding stage may be more difficult to filter, while during longer presentation duration, distractions appearing both in the encoding and delay stages may become increasingly challenging to resist.

Additionally, our results demonstrate that for high-precision memory tasks, longer exposure presentation duration enhance VWM performance. In contrast, for low-precision memory tasks, we did not observe an overall improvement in VWM performance with longer exposure presentation duration, except for a significant increase in accuracy under the full-distraction condition compared to shorter presentation duration. This suggests that the presentation duration of stimulus encoding is critical for forming high-precision memory representations, consistent with previous findings using continuous recall tasks that demonstrate VWM performance improvements with extended encoding time^44^. This finding also aligns with our previously proposed two-phase model of VWM resource allocation, which posits that forming VWM representations involves early and late consolidation stages^36–38^. During early consolidation, individuals form low-precision representations of as many target items as possible. Only after sufficient encoding time can individuals complete early consolidation and enter the late consolidation stage, where high-precision representations are formed as needed by the task. Consequently, extending stimulus encoding time significantly enhances the formation of high-precision representations, whereas for tasks requiring lower memory precision, prolonged exposure has a negligible impact on VWM performance.

Thus, in Experiment 1, by controlling stimulus presentation duration, we observed results that appeared to support seemingly contradictory findings from previous encoding-distraction^13,19–21^ and the studies by Duan, et al.^33^ and Ye, et al.^34^. This underscores the crucial role of stimulus presentation duration in determining whether individuals can effectively resist distractor interference.

Furthermore, it is noteworthy that previous studies by Duan, et al.^33^ and Ye, et al.^34^ found that individuals could relatively easily resist full-distraction, similar to their resistance to encoding-distraction. In their studies, the presentation duration of full-distraction was twice as long as the target presentation. However, in our study, we observed evidence that full-distraction impairs VWM performance, especially during shorter presentation duration. This may be due to the fact that in our study, under short presentation duration, the presentation duration of full-distraction was seven times longer than that of target presentation, whereas under long presentation duration, it was only three times longer. This difference provided participants with relatively longer exposure to distractions under the short presentation condition, increasing susceptibility to impairment from full-distraction. Therefore, the prolonged presence of distractors in our full-distraction condition may account for the inability of some participants to effectively suppress full-distraction.

Although existing findings and our current results consistently support the notion that delay-stage distractors significantly impair VWM performance, these studies often involve distractors that are of the same type as the targets^33,34^. For example, in Experiment 1, in the encoding-distraction condition, participants needed to remember three orientations, they could compare targets and distractors to identify and suppress the irrelevant items during the encoding stage. However, in the delay-distraction condition, when the target orientations disappeared and only distractors remained, participants may have automatically consolidated the new distractor orientations during the delay stage, creating competing VWM representations that impaired performance. Therefore, if distractors are of a different type than the targets (e.g., faces instead of orientations), participants may more effectively filter these heterogeneous distractors, even during the delay stage. However, no previous delay-distraction studies have examined the influence of distractor-target similarity on the delay-distraction effect.

In Experiment 2, we will further explore how stimulus presentation duration affects distractor interference at different stages, while also controlling for the similarity between distractor and target stimuli. This will allow for a more detailed examination of how individuals process and suppress distractors at different stages and how stimulus presentation duration modulates these processes.

## Experiment 2

To further investigate whether the similarity between distractor and target stimuli modulates the effect of presentation duration on VWM performance under different distraction conditions, participants completed a change detection task similar to Experiment 1. Since our research focus was on the impact of stimulus presentation duration on distractors appearing at different stages, we aimed to minimize the influence of presentation duration on the maintenance of VWM representations. Thus, we selected a change detection task for Experiment 2, which is less affected by stimulus duration in terms of VWM performance.

We controlled the duration of stimulus presentation duration and retained the three distraction conditions from Experiment 1: no-distraction, full-distraction, and delay-distraction. Given that the results of the change detection task in Experiment 1 showed no evidence of differences between the no-distraction and encoding-distraction conditions, we did not include encoding-distraction condition in Experiment 2. However, in both the full-distraction and delay-distraction conditions, we introduced two types of distractor stimuli. In the full-orientation-distraction and delay-orientation-distraction conditions, orientation stimuli (same category as the target) were used as distractors. In the full-face-distraction and delay-face-distraction conditions, face stimuli (different category from the target) served as distractors.

### Methods

#### Participants

The sample size for Experiment 2 was similar to that of Experiment 1. A new sample of 29 college students participated in the study in exchange for compensation. However, two participants were excluded from data analyses due to accuracy below chance level (0.5), resulting in a final sample of 27 valid participants (all female; mean age = 20.52, SD = 1.503, age range 18–24 years). All participants reported normal or corrected-to-normal vision, normal color vision, and no history of neurological conditions. Written informed consent was obtained from each participant prior to the study.

### Materials

The stimuli for Experiment 2 included both arrow and face stimuli. The arrow stimuli were identical to those used in Experiment 1, except for an increase in size to match the face stimuli, with each arrow now measuring 2.6° × 1.3° in visual angle. The method of selecting arrow orientations remained consistent with Experiment 1. For the face stimuli, we used the same set as used by Ye, et al.^45^, which consisted of 18 images of neutral male faces selected from the Chinese Facial Affective Picture System CFAPS;^46^. The CFAPS is extensively used in China to investigate human face processing^21,47–51^. All CFAPS images are standardized in terms of size, background, spatial frequency, contrast, brightness, and other physical characteristics. Each image included had a high agreement rate in emotion categorization, with over 70% agreement for each neutral expression. All stimuli were presented randomly within a memory array covering an 11° × 8.2° area centered around the fixation cross. Face stimuli appeared exclusively as task-irrelevant distractors. The distance between any two faces was at least 4.6° (center-to-center). The experiment was programmed using E-Prime software (E-Prime 2.0, Psychology Software Tools, Inc.), and participants were seated in a dark, soundproof room at a viewing distance of 70 cm from a 17-inch screen.

### Procedure

To examine the effect of target encoding time on distraction processing, we manipulated two factors: target presentation duration (short and long) and distraction type (no-distraction, full-orientation-distraction, full-face-distraction, delay-orientation-distraction, and delay-face-distraction).

**Fig. 4:**
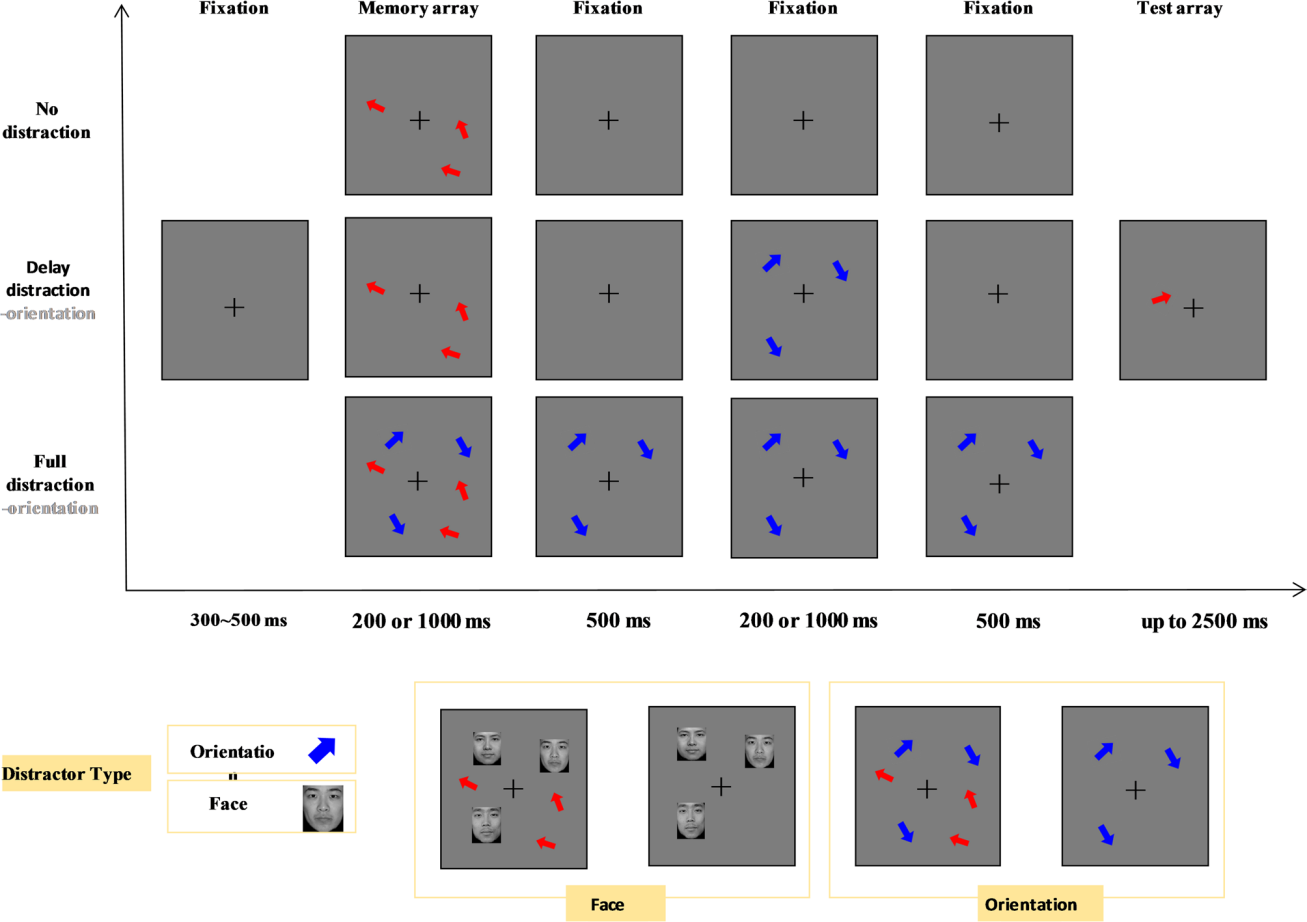
Schematic trial structure of the change detection task used in Experiment 2. There are five distraction conditions: no distraction, full distraction with orientation stimuli, full distraction with face stimuli, delay distraction with orientation stimuli, and delay distraction with face stimuli. Each trial began with a fixation display, followed by a memory array of three red arrows (targets), presented either briefly (200 ms) or for a longer duration (1000 ms). After the memory array, distractors (either blue arrows or grayscale face images from the CFAPS database^46^) appeared during the delay period depending on the condition. In full distraction conditions, distractors were presented during both the memory array and the delay interval. In delay-only conditions, distractors were presented only during the delay phase. No distractors were shown in the baseline condition. A probe arrow then appeared, and participants judged whether the probe arrow matched the orientation of the original target at the same location.The bottom panel provides close-up examples of the two distractor types (orientation vs. face) used in the task.

The trial structure of Experiment 2 is shown in Fig. 4. The procedure was similar to the change detection task in Experiment 1, where participants were required to memorize three target arrows while ignoring other distractor items. The no-distraction condition, full-orientation-distraction condition, and delay-orientation-distraction condition in Experiment 2 were identical to the corresponding no-distraction, full-distraction, and delay-distraction conditions in the Experiment 1 change detection task.

In the newly added full-face-distraction and delay-face-distraction conditions, face stimuli served as distractors. In the full-face-distraction condition, target arrows appeared for either 200 ms (short presentation) or 1000 ms (long presentation) before disappearing. The distractor faces were displayed alongside the target arrows and remained visible during the delay period after the targets disappeared, only disappearing when the test arrow appeared. In the delay-face-distraction condition, after the target arrows disappeared, three distractor faces appeared 500 ms later at random locations outside the target positions for 200 ms (short presentation) or 1000 ms (long presentation). These faces disappeared 500 ms before the test arrow was presented.

Experiment 2 used a within-subject design with factors of presentation duration (short and long) and distraction condition (no-distraction, full-orientation-distraction, full-face-distraction, delay-orientation-distraction, and delay-face-distraction). The change detection task included 48 trials per condition, totaling 480 trials. The entire experiment took approximately 60 min, with 18 practice trials provided to ensure participants understood the procedure.

#### Data analysis

The data analysis approach for Experiment 2 followed that of Experiment 1’s change detection task. The main dependent variable was ACC across each condition. A two-way repeated-measures ANOVA was conducted with presentation duration (short vs. long) and distraction condition (no-distraction vs. full-orientation-distraction, full-face-distraction vs. delay-orientation-distraction vs. delay-face-distraction) as within-subject factors. To further examine the distraction effects of each condition, we conducted planned comparisons using paired t-tests between each distraction condition and the no-distraction baseline, as well as comparisons of the effects of different presentation durations within each distraction condition. We also calculated distraction costs by computing the performance difference between each distraction condition and the no-distraction baseline as Experiment 1. To further characterize the pattern of distraction costs, we conducted a three-way repeated-measures ANOVA with presentation duration (short vs. long), distraction condition (full vs. delay), and distractor type (orientation vs. face) as within-subject factors. Planned comparisons were also conducted to examine differences in distraction cost across conditions and presentation duration. Partial eta squared (η^2^_p_) was reported as the effect size estimate for the ANOVA results. Effect sizes for the t-tests were reported as Cohen’s d. Additionally, Bayes Factor analyses were performed to determine whether the t-test results supported the alternative hypothesis over the null hypothesis^43^.

### Results

#### Accuracy

**Fig. 5:**
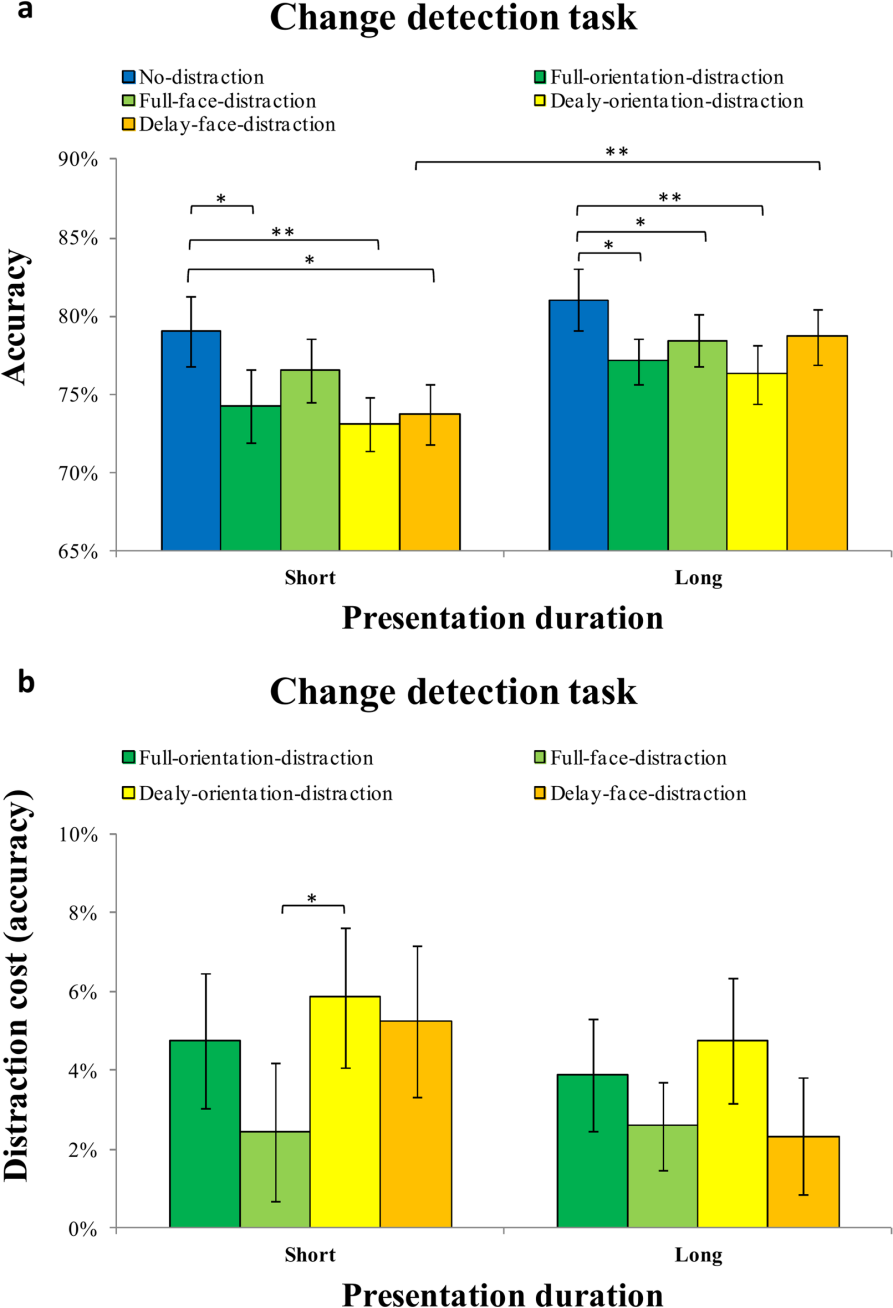
Results of the change detection task in Experiment 2. (**a**) Mean accuracy in the change detection task as a function of presentation duration (short vs. long) and distraction condition (no-distraction, full-orientation-distraction, full-face-distraction,. delay-orientation-distraction, delay-face-distraction) in Experiment 2. (**b**) Distraction cost (i.e., accuracy difference from the no-distraction condition) across distraction conditions (no-distraction, full-orientation-distraction, full-face-distraction,. delay-orientation-distraction, delay-face-distraction) for each presentation duration. Error bars represent ± 1 standard error of the mean (SEM). *p < 0.05; **p < 0.01; ***p < 0.001;

The mean accuracy for each distraction condition (no-distraction vs. full-orientation-distraction vs. full-face-distraction vs. delay-orientation-distraction vs. delay-face-distraction) under short or long presentation duration is presented in Fig. 5a. The ANOVA revealed a significant main effect of presentation duration, F (1,26) = 11.703, *p* = 0.002, η^2^_p_ = 0.310, and a significant main effect of the distraction condition, F (4,104) = 5.772, *p* < 0.001, η^2^_p_ = 0.182. However, no significant interaction was found between the presentation duration and distraction condition, F (4,104) = 0.634, *p* = 0.639, η^2^_p_ = 0.024.

Planned pairwise comparisons indicated that, under the short presentation duration, accuracy in the no-distraction condition was significantly higher than in the full-orientation-distraction condition, t(26) = 2.780, *p* = 0.010, Cohen’s d = 0.535, BF_10_ = 4.676, delay-orientation-distraction condition, t(26) = 3.271, *p* = 0.003, Cohen’s d = 0.629, BF_10_ = 12.967, and the delay-face-distraction condition, t(26) = 2.746, *p* = 0.011, Cohen’s d = 0.528, BF_10_ = 4.369. However, no significant difference in accuracy was observed between the no-distraction and full-face-distraction conditions, t(26) = 1.091, *p* = 0.175, Cohen’s d = 0.269, BF_10_ = 0.485, suggesting that short-term exposure to face-based distraction during encoding did not impair memory performance. Under the long presentation duration, accuracy in the no-distraction condition remained significantly higher than in the delay-orientation-distraction condition, t(26) = 2.993, *p* = 0.006, Cohen’s d = 0.576, BF_10_ = 7.208, the full-orientation-distraction condition, t(26) = 2.700, *p* = 0.012, Cohen’s d = 0.520, BF_10_ = 3.989, and the full-face-distraction condition, t(26) = 2.302, *p* = 0.030, Cohen’s d = 0.443, BF_10_ = 1.896, but showed no significant differences with the delay-face-distraction condition, t(26) = 1.582, *p* = 0.126, Cohen’s d = 0.304, BF_10_ = 0.614. This pattern suggests that, although extended encoding time generally mitigated the effects of distraction, delayed orientation distractors continued to impair performance.

Additionally, accuracy for the long presentation duration was significantly higher than that for the short presentation duration only in the delay-face-distraction condition, t(26) = 3.037, *p* = 0.005, Cohen’s d = 0.584, BF_10_ = 7.894. However, no significant differences in accuracy were found between long and short presentation durations in the no-distraction condition, t(26) = 1.462, *p* = 0.156, Cohen’s d = 0.281, BF_10_ = 0.526; full-orientation-distraction condition, t(26) = 1.566, *p* = 0.129, Cohen’s d = 0.301, BF_10_ = 0.602; full-face-distraction condition, t(26) = 1.134, *p* = 0.267, Cohen’s d = 0.218, BF_10_ = 0.364; or delay-orientation-distraction condition, t(26) = 1.971, *p* = 0.059, Cohen’s d = 0.379, BF_10_ = 1.086. These findings indicate that the facilitating effect of longer encoding time on accuracy was limited and condition-specific.

#### Distraction cost (ACC)

The mean distraction cost (ACC) for each distraction condition (encoding-distraction, full-distraction, and delay-distraction) under short and long presentation durations is shown in Fig. 5b. The ANOVA on distraction cost (ACC) revealed a significant main effect of the distractor type, F (1,26) = 6.865, *p* = 0.014, η^2^_p_ = 0.209. The distraction cost was significantly lower for face distractors (M = 0.0316, SD = 0.059) than for orientation distractors (M = 0.048, SD = 0.053), t(26) = 2.62, *p* = 0.014, Cohen’s d = 0.504, BF_10_ = 3.417. However, there was no significant main effect of presentation duration, F (1,26) = 0.625, *p* = 0.436, η^2^_p_ = 0.023, nor of distraction condition, F (1,26) = 1.477, *p* = 0.235, η^2^_p_ = 0.054. Furthermore, none of the two-way interactions reached significance: presentation duration × distraction condition, F (1,26) = 1.463, *p* = 0.237, η^2^_p_ = 0.053; presentation duration × distractor type, F (1,26) = 0.049, *p* = 0.826, η^2^_p_ = 0.002; or distraction condition × distractor type, F (1,26) = 0.028, *p* = 0.869, η^2^_p_ = 0.001. The three-way interaction among presentation duration, distraction condition, and distractor type was also not significant, F (1,26) = 0.798, *p* = 0.380, η^2^_p_ = 0.030.

Planned pairwise comparisons were conducted to further examine distraction cost differences under each presentation duration condition. Under short presentation duration, a significant difference emerged between the delay-orientation-distraction and full-face-distraction conditions, with the former eliciting greater distraction cost, t(26) = 2.41, *p* = 0.023, Cohen’s d = 0.464, BF_10_ = 2.299. However, all other pairwise comparisons within the short presentation duration condition failed to reach significance (all ps > 0.093), indicating no reliable differences in distraction cost among the remaining combinations of distractor type and timing.

Under long presentation duration, no significant differences in distraction cost were found across any of the distraction conditions (all ps > 0.202), suggesting that increasing encoding time may mitigate distraction effects regardless of distractor type or timing.

In addition, comparisons across presentation durations revealed no significant differences in distraction cost for any individual condition. Specifically, distraction costs were statistically comparable between short and long presentation durations in all conditions, with all ps > 0.133, indicating that extending presentation duration did not systematically reduce distraction-related performance costs in any of the examined conditions.

### Discussion

In Experiment 2, we found that under short presentation duration, both same-category and different-category distractors presented as delay-distractions impaired VWM performance, whereas only same-category distractors presented as full-distractions had a detrimental effect. This indicates that when stimulus presentation is brief, delay-stage distractors, regardless of their type, impair VWM performance, while continuous distractors spanning both encoding and delay stages do not impact performance if they are of a different category. This result may arise because, under short presentation durations, memory representations are less consolidated and therefore more vulnerable to sudden distractor interference during the delay stage. Conversely, when distractors persist through both encoding and delay stages, especially for different-category distractors, participants may suppress such interference starting from the encoding phase, thereby mitigating its impact on VWM maintenance.

Additionally, with long presentation duration, both same-category and different-category distractors presented as full-distractions impaired VWM performance, while only same-category distractors presented as delay-distractions had a negative effect. This suggests that when stimuli are presented for a longer duration, all types of continuous distractions spanning encoding and delay stages disrupt VWM performance. However, when distractors appear only during the delay stage, only same-category distractors continue to impair performance, while different-category distractors no longer have an effect. This differential effect of delay-distraction based on distractor-target similarity aligns with our hypothesis: when the distractor type is distinctly different from the target type, the delay-distraction effect diminishes. This may be because with extended presentation durations, participants have already formed stable VWM representations. This finding provides the first evidence, following the work of Duan, et al.^33^ and Ye, et al.^34^, that participants can resist the negative impact of delay-stage distractors. In the full-distraction condition, however, resource competition between distractors and target stimuli during encoding can impair VWM maintenance. When distractors appear solely during the delay stage, participants with stable VWM representations may resist sudden interference unless the distractors are of the same category, which may lead to automatic consolidation of the distractors, thereby impairing VWM performance. Conversely, different-category distractors may be more easily identified and suppressed without requiring additional resources.

Furthermore, our findings indicate that when face distractors are presented during the delay stage, VWM performance is more severely impaired under short presentation durations compared to long durations. This further supports the notion that longer encoding and consolidation times for target stimuli can help participants better resist the negative effects of heterogeneous distractors during the delay stage. Overall, heterogeneous distractors tend to have a weaker detrimental effect on VWM maintenance compared to homogeneous distractors, likely because participants more readily identify and suppress heterogeneous distractors.

It is noteworthy that we selected neutral faces as heterogeneous distractors. Previous research has shown that faces capture attention more efficiently than other meaningful objects^52–55^. Therefore, although face distractors are of a different category, they may still attract more attention and potentially cause greater disruption than orientation distractors. However, if participants can resist the negative impact of heterogeneous distractors even under these conditions, this suggests that stable VWM representations can indeed protect against such interference. This further supports the idea that once stable VWM representations are formed, participants can resist delay-stage distractions.

It is also important to consider that face stimuli are not only categorically distinct from orientation stimuli, but also perceptually more complex. Thus, the observed distractor effects in Experiment 2 could reflect differences in processing load rather than stimulus category per se. To disentangle these possibilities, it is necessary to examine whether the perceptual load imposed by distractors independently influences distractor filtering mechanisms.

To address this, in Experiment 3 we used distractors that were all of the same type as the targets (i.e., orientation stimuli), while systematically manipulating the number of distractors to vary their perceptual load. This allowed us to isolate the effects of distractor complexity from stimulus type, enabling a more fine-grained examination of how individuals process and suppress distractors at different stages of VWM, and how these processes are modulated by stimulus presentation duration.

## Experiment 3

To promote effective control over perceptual load, we aimed to encourage participants to engage in more fine-grained perceptual processing even for orientation stimuli, which are generally considered less complex than faces. To this end, Experiment 3 used a continuous recall task, which typically places higher demands on perceptual precision than change detection tasks.

Moreover, to eliminate any confounding influence of distractor duration—particularly in the full-distraction condition, where distractors would remain onscreen much longer than in the delay-distraction condition—we did not include the full-distraction condition in Experiment 3. Instead, we compared only the encoding- and delay-distraction conditions, with distractors in both conditions presented for the same duration.

We manipulated the number of distractors to vary perceptual load: three distractors (as in Experiments 1 and 2) constituted the low perceptual load condition, and five distractors constituted the high perceptual load condition. This manipulation allowed us to assess whether perceptual load modulates the impact of distraction at different stages of VWM.

### Methods

#### Participants

Twenty-four undergraduate students (23 female, 1 male; mean age = 20.63 years, SD = 1.38, age range: 18–24 years) participated in the experiment in exchange for monetary compensation. All participants reported normal or corrected-to-normal vision, normal color vision, and no history of neurological or psychiatric disorders. Written informed consent was obtained from each participant prior to participation.

### Materials

The stimuli and apparatus were identical to those used in the continuous recall task in Experiment 1. The only difference was that in the high distraction load condition, five distractor orientations were presented simultaneously at random locations on the screen during the distractor display.

### Procedure

To investigate how the duration of target encoding modulates the processing of distracting information, we manipulated two within-subject factors: presentation duration (short vs. long) and distraction condition (no-distraction, encoding-low-distraction, encoding-high-distraction, delay-low-distraction, and delay-high-distraction).

**Fig. 6:**
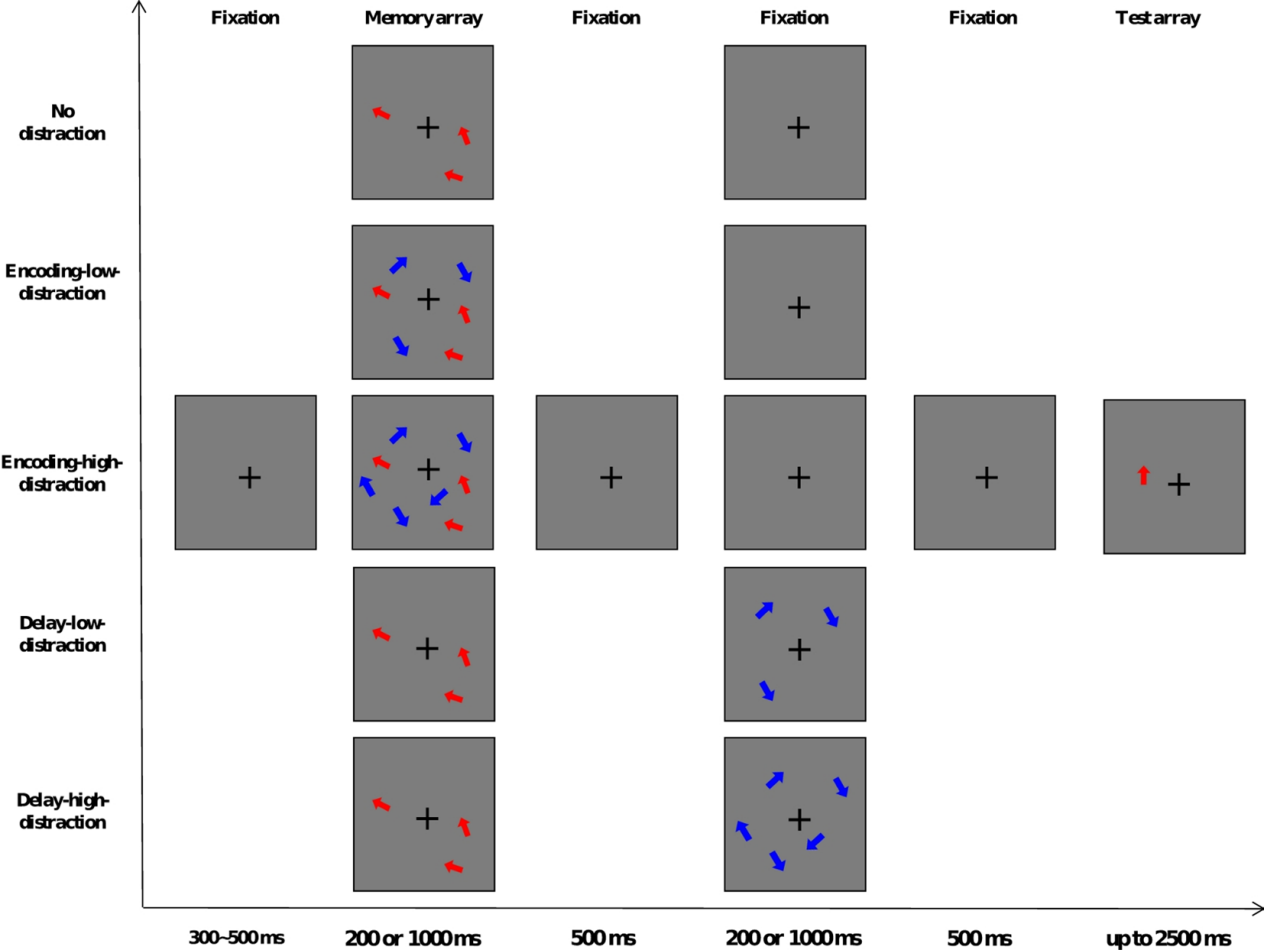
Schematic trial structure of the continuous recall task in Experiment 3. There are five distraction conditions: no distraction, encoding-stage distraction (low and high load), and delay-stage distraction (low and high load). Each trial began with a fixation display, followed by a memory array of three red arrows (targets), presented either for a short duration (200 ms) or a long duration (1000 ms), depending on the condition. In the distraction conditions, blue arrows (distractors) appeared during the memory array (encoding distraction), the delay interval (delay distraction), or not at all (no distraction). The number of distractors was manipulated to create low-load (3 distractors) and high-load (5 distractors) conditions. After a retention interval, a single probe arrow appeared, and participants were instructed to adjust its orientation to match the target previously shown at the same location.

The overall trial structure is illustrated in Fig. 6. The procedure closely followed that of the continuous recall task in Experiment 1, in which participants memorized the orientations of three target arrows while ignoring concurrent or subsequent distractors. The no-distraction, encoding-low-distraction, and delay-low-distraction conditions were identical to the corresponding conditions (no-distraction, encoding-distraction, and delay-distraction condition) in the continuous recall task of Experiment 1.

Two new conditions were introduced to examine the effects of increased distraction load: encoding-high-distraction and delay-high-distraction. In these conditions, the number of distractors was increased from three to five orientation stimuli. In the encoding-high-distraction condition, five orientation distractors appeared alongside the targets and disappeared simultaneously with them, after either 200 ms (short duration) or 1000 ms (long duration). In the delay-high-distraction condition, after the targets disappeared, five orientation distractors were presented 500 ms later at random non-target locations for 200 ms (short duration) or 1000 ms (long duration), followed by a 500 ms blank interval before the test display.

Experiment 3 used a within-subject design with factors of presentation duration (short vs. long) and distraction condition (no-distraction vs. encoding-low-distraction vs. encoding-high-distraction vs. delay-low-distraction vs. delay-high-distraction). The continuous recall task included 48 trials per condition, totaling 480 trials. The entire experiment took approximately 60 min, with 18 practice trials provided to ensure participants understood the procedure.

#### Data analysis

The data analysis approach for Experiment 3 followed that of Experiment 1’s continuous recall task. The main dependent variable was offset across each condition. A two-way repeated-measures ANOVA was conducted with presentation duration (short vs. long) and distraction condition (no-distraction, encoding-low-distraction, encoding-high-distraction, delay-low-distraction, and delay-high-distraction) as within-subject factors. To further examine the distraction effects of each condition, we conducted planned comparisons using paired t-tests between each distraction condition and the no-distraction baseline, as well as comparisons of the effects of different presentation durations within each distraction condition. We also calculated distraction costs by computing the performance difference between each distraction condition and the no-distraction baseline as Experiment 1. To further characterize the pattern of distraction costs, we conducted a three-way repeated-measures ANOVA with presentation duration (short vs. long), distraction condition (encoding vs. delay), and distraction load (low vs. high) as within-subject factors. Planned comparisons were also conducted to examine differences in distraction cost across conditions and presentation duration. Partial eta squared (η^2^_p_) was reported as the effect size estimate for the ANOVA results. Effect sizes for the t-tests were reported as Cohen’s d. Additionally, Bayes Factor analyses were performed to determine whether the t-test results supported the alternative hypothesis over the null hypothesis^43^.

### Results

#### Offset

**Fig. 7:**
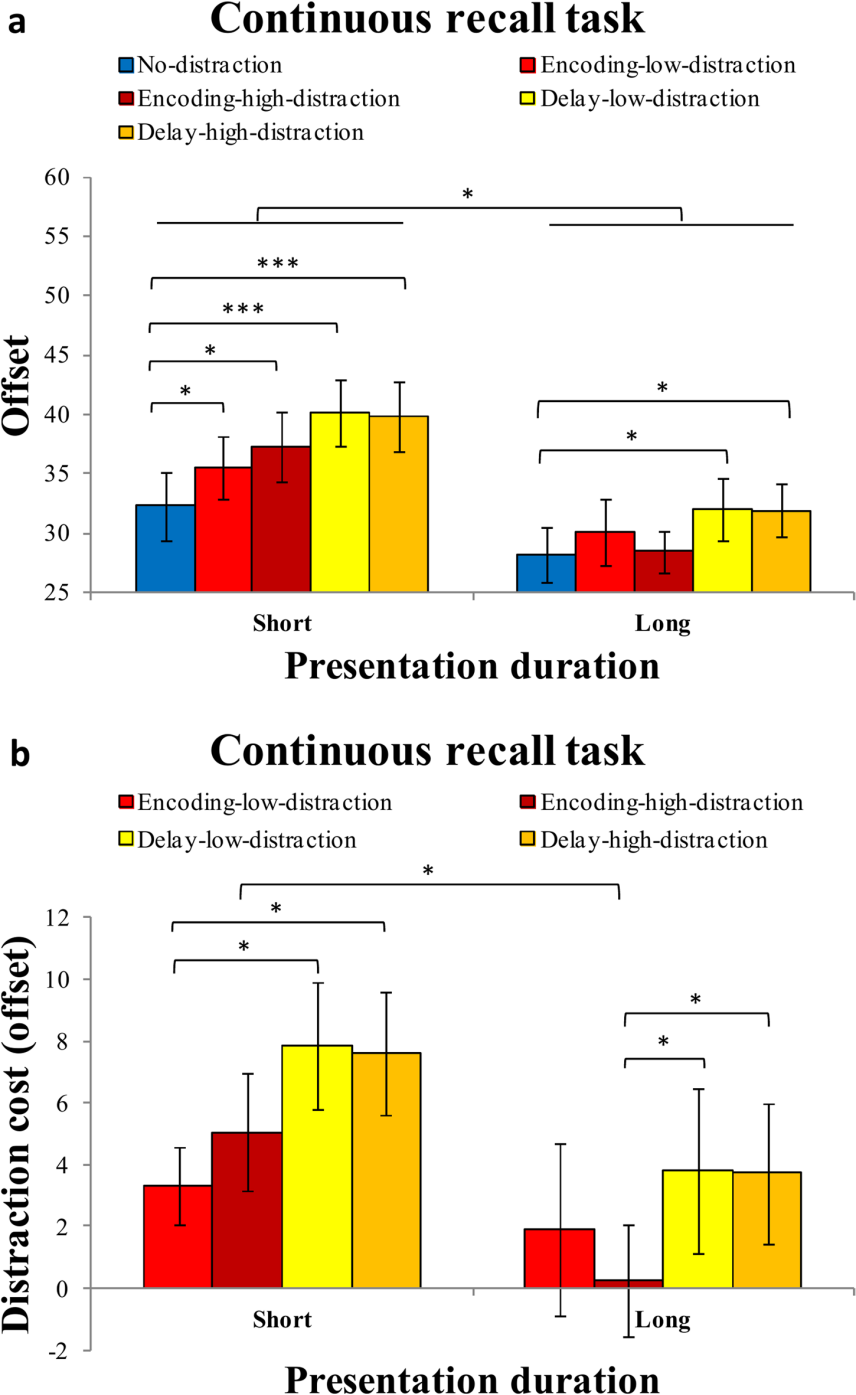
Results of the continuous recall task in Experiment 3. (**a**) Mean recall error (offset in degrees) as a function of presentation duration (short vs. long) and distraction condition (no-distraction, encoding-low-distraction, encoding-high-distraction, delay-low-distraction, delay-high-distraction) in the continuous recall task of Experiment 3. (**b**) Distraction cost (i.e., offset relative to the no-distraction condition) under each distraction condition (encoding-low-distraction, encoding-high-distraction, delay-low-distraction, delay-high-distraction) for both short and long presentation durations. Error bars represent ± 1 standard error of the mean (SEM). *p < 0.05; ***p < 0.001.

The mean offset for each distraction condition (no-distraction, encoding-low-distraction, encoding-high-distraction, delay-low-distraction, and delay-high-distraction) under short or long presentation duration is presented in Fig. 7a. The ANOVA revealed a significant main effect of presentation duration, F (1,23) = 48.887, *p* < 0.001, η^2^_p_ = 0.680, and a significant main effect of the distraction condition, F (4,92) = 7.029, *p* < 0.001, η^2^_p_ = 0.234. However, no significant interaction was found between the presentation duration and distraction condition, F (4,92) = 1.410, *p* = 0.245, η^2^_p_ = 0.058.

Planned pairwise comparisons revealed that, under the short presentation duration, the offset in the no-distraction condition was significantly lower than in the encoding-low-distraction condition, t(23) = 2.609, *p* = 0.016, Cohen’s d = 0.532, BF_10_ = 3.324; the encoding-high-distraction condition, t(23) = 2.661, *p* = 0.014, Cohen’s d = 0.543, BF_10_ = 3.665; the delay-low-distraction condition, t(23) = 3.802, *p* < 0.001, Cohen’s d = 0.776, BF_10_ = 37.744, and the delay-highdistraction condition, t(23) = 3.795, *p* < 0.001, Cohen’s d = 0.775, BF_10_ = 37.246. These results suggest that distraction—whether occurring during encoding or maintenance—reduced VWM performance when exposure duration was limited. However, under the long presentation duration, the result pattern changed. The offset in the no-distraction condition was no longer significantly different from either the encoding-low-distraction condition, t(23) = 1.179, *p* = 0.250, Cohen’s d = 0.241, BF_10_ = 0.399, or the encoding-high-distraction condition, t(23) = 0.182, *p* = 0.857, Cohen’s d = 0.037, BF_10_ = 0.218. In contrast, delay-stage distraction continued to impair performance even under extended exposure: the offset in the no-distraction condition was significantly lower than in the delay-low-distraction condition, t(23) = 2.280, *p* = 0.032, Cohen’s d = 0.465, BF_10_ = 1.846, and the delay-high-distraction condition, t(23) = 2.500, *p* = 0.020, Cohen’s d = 0.510, BF_10_ = 2.721.

Additionally, the offset for the long presentation duration was significantly lower than that for the short presentation duration across all conditions: no-distraction, t(23) = 2.657, *p* = 0.014, Cohen’s d = 0.540, BF_10_ = 3.570; encoding-low-distraction, t(23) = 4.776, *p* < 0.001, Cohen’s d = 0.975, BF_10_ = 325.307; encoding-high-distraction, t(23) = 4.633, *p* < 0.001, Cohen’s d = 0.946, BF_10_ = 236.790; delay-low-distraction, t(23) = 3.313, *p* = 0.003, Cohen’s d = 0.676, BF_10_ = 13.363, and delay-high-distraction, t(23) = 4.541, *p* < 0.001, Cohen’s d = 0.927, BF_10_ = 192.494. These results confirm that longer presentation durations consistently enhance VWM performance, regardless of distraction timing or load.

#### Distraction cost (offset)

Figure 7b displays the mean distraction cost (offset) for each condition—encoding-low-distraction, encoding-high-distraction, delay-low-distraction, and delay-high-distraction—under short and long presentation durations. A repeated-measures ANOVA revealed a significant main effect ofdistraction condition, F (1,23) = 12.658, *p* = 0.002, η^2^_p_ = 0.355. The distraction cost in the encoding-distraction condition (M = 2.629, SD = 4.939) was significantly lower than in the delay-distraction condition (5.745 ± 5.87), t(23) = 3.558, *p* = 0.002, Cohen’s d = 0.726, BF_10_ = 22.364. A significant main effect of presentation duration was also found, F (1,23) = 5.348, *p* = 0.030, η^2^_p_ = 0.189, with lower distraction costs under long presentation duration (M = 2.419, SD = 5.486) compared to short duration (M = 0.955, SD = 6.899), t(23) = 2.313, *p* = 0.03, Cohen’s d = 0.472, BF_10_ = 1.954. However, there was no significant main effect of distraction load, F (1,23) = 0.004, *p* = 0.950, η^2^_p_ = 0. Furthermore, none of the two-way interactions reached significance: presentation duration × distraction condition, F (1,23) = 0.305, *p* = 0.586, η^2^_p_ = 0.013; presentation duration × distraction load, F (1,23) = 0.582, *p* = 0.453, η^2^_p_ = 0.025; or distraction condition × distraction load, F (1,23) = 0.012, *p* = 0.912, η^2^_p_ = 0.001. The three-way interaction among presentation duration, distraction condition, and distraction load was also not significant, F (1,23) = 1.050, *p* = 0.316, η^2^_p_ = 0.044.

Under short presentation duration, there was no significant difference in distraction cost between the encoding-low-distraction and encoding-high-distraction conditions, t(23) = 1.12, *p* = 0.273, Cohen’s d = 0.229, BF_10_ = 0.377, indicating that increasing the distraction load during encoding did not further impair performance. Similarly, distraction cost did not differ between the delay-low-distraction and delay-high-distraction conditions, t(23) = 0.12, *p* = 0.903, Cohen’s d = 0.025, BF_10_ = 0.216, suggesting that distraction load during the delay period had minimal impact. When comparing across temporal stages, no significant difference was observed between the encoding-high-distraction and delay-low-distraction conditions, t(23) = 1.35, *p* = 0.191, Cohen’s d = 0.275, BF_10_ = 0.478, nor between the encoding-high-distraction and delay-high-distraction conditions, t(23) = 1.25, *p* = 0.226, Cohen’s d = 0.254, BF_10_ = 0.427. However, a significant difference emerged when comparing the encoding-low-distraction condition to both delay conditions: distraction cost in the encoding-low-distraction condition was significantly lower than in the delay-low-distraction condition, t(23) = 2.23, *p* = 0.036, Cohen’s d = 0.455, BF_10_ = 1.690, and also lower than in the delay-high-distraction condition, t(23) = 2.51, *p* = 0.020, Cohen’s d = 0.512, BF_10_ = 2.773. These results suggest that, under time pressure, even minimal distraction during the delay period is more disruptive to memory performance than low-level distraction during encoding.

Under long presentation duration, the pattern of results was similar, though generally weaker. Distraction cost did not significantly differ between the encoding-low-distraction and encoding-high-distraction conditions, t(23) = 0.84, *p* = 0.409, Cohen’s d = 0.172, BF_10_ = 0.295, nor between the delay-low-distraction and delay-high-distraction conditions, t(23) = 0.04, *p* = 0.969, Cohen’s d = 0.008, BF_10_ = 0.215. Furthermore, no significant differences were observed between the encoding-low-distraction and delay-low-distraction conditions, t(23) = 1.35, *p* = 0.192, Cohen’s d = 0.275, BF_10_ = 0.477, or between the encoding-low-distraction and delay-high-distraction conditions, t(23) = 1.07, *p* = 0.298, Cohen’s d = 0.217, BF_10_ = 0.357. However, significant difference was found when comparing encoding-high-distraction to the delay conditions: distraction cost was significantly lower in the encoding-high-distraction condition than in both the delay-low-distraction condition, t(23) = 2.08, *p* = 0.049, Cohen’s d = 0.425, BF_10_ = 1.331, and the delay-high-distraction condition, t(23) = 2.43, *p* = 0.023, Cohen’s d = 0.496, BF_10_ = 2.406. These findings indicate that even with extended encoding time, distraction during the delay period remains more detrimental to performance than distraction occurring during encoding.

In addition, distraction cost did not significantly differ between short and long presentation durations for encoding-low-distraction condition, t(23) = 0.732, *p* = 0.472, Cohen’s d = 0.149, BF_10_ = 0.274; delay-low-distraction, t(23) = 1.526, *p* = 0.141, Cohen’s d = 0.312, BF_10_ = 0.594; or delay-high-distraction, t(23) = 1.803, *p* = 0.085, Cohen’s d = 0.368, BF_10_ = 0.866. However, distraction cost in the encoding-high-distraction condition was significantly lower under long presentation duration compared to short presentation duration, t(23) = 2.47, *p* = 0.021, Cohen’s d = 0.504, BF_10_ = 2.571. This finding implies that longer encoding durations may allow participants to better resist or compensate for the disruptive influence of high-load distractors during encoding.

### Disscusion

Experiment 3 aimed to clarify how distraction stage (encoding vs. delay) and perceptual load (low vs. high) influence VWM when encoding duration varies (short vs. long). Results showed that neither the stage at which distractors were presented nor their perceptual load significantly altered distraction magnitude. Across both short and long exposure durations, delay-stage distractors consistently produced greater memory disruption compared to encoding-stage distractors. Importantly, increasing the number of distractors from three (low load) to five (high load) did not intensify the disruptive effect. Together with Experiments 1 and 2, these findings support a robust asymmetry in distractor processing across VWM stages. Specifically, any interference occurring after initial consolidation—even briefly—appears to corrupt the stored representation similarly, irrespective of distractor quantity. We interpret this pattern as evidence that distraction after consolidation targets representations already stored in a limited-capacity memory system, rendering the system unable to accommodate additional irrelevant information, regardless of its quantity. Consequently, these results argue against our earlier hypothesis, which attributed the different distraction patterns observed in Experiment 2’s orientation and face distractor conditions to differences in perceptual load.

Under short exposure duration (200 ms), both low- and high-load encoding distractors significantly increased memory errors. Conversely, when participants had longer exposure duration (1000 ms), encoding-stage distractors—regardless of load—no longer impaired performance, and results were statistically equivalent to the no-distraction baseline. This replicates the pattern observed in Experiment 1, highlighting that sufficient consolidation time allows individuals to proactively filter out distractions presented during encoding. Nevertheless, irrespective of encoding duration, participants consistently struggled to filter distractions appearing during the delay period, indicating that delay-stage interference is more challenging to resist, likely due to disruptions targeting consolidated representations directly.

## Genernal discussion

Across three experiments, we systematically examined how different types of distraction—encoding-stage, full-stage, and delay-stage—affect VWM performance under varying encoding durations. In Experiment 2, we manipulated the category of distractors (target-congruent vs. target-incongruent), while in Experiment 3, we manipulated perceptual load by varying the number of distractor items. Crucially, several distraction conditions were directly identital across experiments. For example, Experiments 1 and 2 shared identical distraction conditions in the change detection task (full-distraction and delay-distraction), while Experiments 1 and 3 included matched conditions in the continuous recall task (encoding-distraction and delay-distraction). To provide a more robust statistical assessment, we pooled data across these matched conditions in the supplementary materials (see Supplementary Materials for full analyses).

These analyses revealed that that in the change detection task, encoding-stage distractors did not significantly impair VWM performance under either short or long exposure durations. In contrast, both full-stage and delay-stage distractors led to reliable performance costs, regardless of encoding duration. These results suggest that once encoding is complete VWM representations remain vulnerable to interference throughout the delay interval.

In the continuous recall task, a different pattern emerged. Distraction effects were strongly modulated by encoding time. Under short encoding duration, all types of distractors significantly impaired memory performance. However, with longer encoding duration, only full-stage and delay-stage distractors continued to produce performance deficits, while encoding-stage distractors no longer had a significant impact. These findings suggest that longer exposure allows for more robust consolidation and proactive filtering during encoding, but once distractors are introduced after consolidation, they remain disruptive regardless of initial encoding quality.

These pooled findings point to a notable distinction: while encoding-stage distraction can be countered through sufficient consolidation time or task-specific strategies, delay-stage distraction tends to remain disruptive regardless of the encoding quality. This pattern underscores the unique vulnerability of the maintenance phase in VWM and suggests that post-consolidation interference may directly compromise the integrity of already-formed memory traces.

More broadly, our results support the view that VWM consolidation is best characterized as a temporally dynamic, two-stage process^36–38^. In the initial (early consolidation) stage, brief encoding periods appear sufficient to generate low-precision representations, adequate for tasks requiring the detection of relatively large changes (e.g., change detection). However, these early-stage representations remain highly vulnerable to interference if more fine-grained details must be maintained, as required by tasks such as continuous recall. Longer encoding durations enable a transition into a subsequent (late consolidation) stage, where high-precision representations are stabilized and more effectively protected against interference from concurrently presented distractors.

Our results further clarify the nature of delay-stage distractor interference. They demonstrate that once consolidation is complete—regardless of the precision level of stored representations—any subsequent distractor input competes directly with established memory representations, consistently degrading performance across task types. This finding implies that late-stage VWM representations, although relatively robust against early-stage interference, remain constrained by fixed capacity limits. Thus, distractors presented after consolidation may inevitably degrade or displace existing representations, reflecting resource competition within a capacity-limited memory store.

Additionally, we observed that participants’ ability to resist distractors spanning both encoding and delay intervals (i.e., full-stage distraction) depended heavily on the relative timing and duration of distractor exposure. When distractors persisted substantially longer than target stimuli—particularly under brief target presentations—participants struggled to effectively suppress irrelevant information, possibly due to extended demands on attentional control mechanisms. This underscores the critical interplay between consolidation duration and the timing of interference, with prolonged distractor presence amplifying interference effects through sustained demands on cognitive resources.

We also recognize an alternative interpretation to our results. According to the attentional competition hypothesis^56,57^, longer encoding durations might benefit memory performance not only by enhancing consolidation but also by reducing attentional competition between target and distractor stimuli during encoding. Specifically, under short-duration conditions, distractors may more effectively capture attention, resulting in incomplete or disrupted encoding of target items. In contrast, longer encoding durations could mitigate this attentional competition, providing participants adequate time to disengage attention from distractors and reorient toward targets, thereby facilitating complete and robust encoding of relevant items. Such attentional disengagement processes would enhance the resilience of memory representations against interference from encoding-stage and full-stage distractions, consistent with our findings. Another possible account is that sufficient encoding duration allows some representations to be transferred into a passive state, which renders them less vulnerable to concurrent distractors^58–63^. This mechanism may explain why encoding-stage interference diminishes when longer exposure enables such a protective transition.

Furthermore, the suppression mechanisms operating during encoding and delay stages may differ fundamentally. Suppression during encoding likely involves primarily attentional filtering mechanisms, wherein participants proactively prevent distractors from entering VWM, relying on mechanisms indexed by electrophysiological components such as the Pd and CDA^13,19^. In contrast, suppression during the delay stage may rely predominantly on memory maintenance mechanisms, which involve either active inhibition or the removal of irrelevant representations that have already been encoded into VWM^23,64^. Consequently, delay-stage suppression processes may be more cognitively demanding, requiring sustained attentional resources and active maintenance of suppression signals throughout the delay interval. Future studies employing electrophysiological or neuroimaging approaches could explicitly test these hypotheses by separately characterizing the neural signatures associated with distractor suppression at distinct working memory stages.

Although our current findings cannot definitively distinguish between these complementary accounts—consolidation quality, attentional competition, and distinct suppression mechanisms—together they underscore the critical importance of encoding duration and memory stage in modulating distractor interference. Future work should systematically manipulate factors such as spatial-temporal properties of distractors, distractor similarity to memory targets, and explicit task demands, combined with neural measures sensitive to attention and memory processes. Such comprehensive approaches promise deeper insights into the dynamic interplay among attention, encoding processes, consolidation duration, and memory maintenance mechanisms, thereby further advancing our understanding of VWM capacity limitations and distractor suppression dynamics.

In addition to the main findings from Experiment 1, the results from Experiment 2 offer further insight into how target-distractor similarity and distraction stage jointly modulate VWM performance. Under short presentation durations, we observed that delay-stage distractions impaired memory performance regardless of distractor type, while full-stage distraction effects were evident only when distractors were of the same category as the targets. These results suggest that brief encoding leaves memory traces susceptible to sudden interference during the delay phase, particularly when attentional resources have not fully disengaged from distractor content. However, when distractors persist throughout the encoding and delay phases, participants may be able to initiate early suppression processes—especially for dissimilar distractors—thus reducing their downstream impact on memory. Under longer presentation durations, full-stage distractions from both same- and different-category distractors reliably impaired performance, whereas delay-stage interference was only observed when distractors matched the target category. These findings suggest that stable representations formed under extended encoding durations are generally more resilient to post-encoding interference, particularly when distractors are perceptually distinct. Taken together, the data from Experiment 2 point to an important interaction between consolidation time and distractor similarity, supporting the idea that category-level overlap between targets and distractors increases the likelihood of interference, even when representations are otherwise robust.

Experiment 3 further clarified how distraction stage and perceptual load interact with encoding duration to shape distraction susceptibility in VWM. While we initially hypothesized that higher perceptual load would amplify distraction costs—particularly under short encoding durations—our results did not support this prediction. Instead, distraction magnitude was determined primarily by when the interference occurred, not by how many distractors were present. Across both encoding durations, delay-stage distractors consistently led to greater performance impairment than encoding-stage distractors, regardless of distractor load. These results suggest that even brief interference introduced after initial consolidation may corrupt fragile memory traces stored in a capacity-limited system, with little added cost for increasing the number of irrelevant stimuli. In other words, once post-encoding interference reaches the memory system, its disruptive effect may saturate, consistent with the idea that maintenance-stage suppression operates under different constraints than early attentional filtering. These findings challenge the view that perceptual load alone accounts for differences in distraction patterns across stimulus types and instead reinforce a temporally grounded model of distractor interference in VWM.

We also noted that participants’ performance in the change detection task appeared numerically better in Experiment 2 compared to Experiment 1. To examine this difference, we directly compared the no-distraction condition across Experiments 1 and 2—conditions that were identical in both tasks. The analysis revealed no statistically significant difference in the no-distraction condition performance between the two experiments (*p* = 0.143), indicating that participants performed comparably under baseline conditions (see Supplementary Materials for detailed results).

One possible concern, however, is that participants in Experiment 1 always completed the continuous recall task before the change detection task. This raises the possibility that fatigue may have contributed to diminished performance in the later task. To test this, we divided all trials of the change detection task in Experiment 1 into four sequential blocks and examined whether accuracy declined across blocks. If fatigue were present, performance would be expected to deteriorate over time. However, the data revealed no evidence of such decline (see Supplementary Materials), indicating that task order did not produce fatigue-related impairment. On the contrary, participants’ performance actually improved across blocks and eventually stabilized, reflecting a practice effect rather than fatigue. Such improvements are common in VWM paradigms, especially when participants must develop effective encoding and response strategies. Importantly, our experimental design used randomized within-subject manipulation of both presentation duration and distraction conditions. This design feature mitigates concerns about the influence of practice or fatigue effects on the key findings, ensuring that the reported effects reflect genuine differences in memory processing at distinct temporal stages, rather than being confounded by task sequence or participant adaptation.

Beyond the main findings on distraction stage and presentation duration, it is worth considering the potential influence of participant strategies, particularly under conditions with limited encoding time. One plausible concern is that, when faced with high task demands (e.g., continuous recall under brief presentation durations), participants might rely on simplified strategies such as categorical encoding or ensemble averaging of target features. However, additional analyses provided no evidence supporting these alternatives (see Supplementary Materials, Figure S1-2, for details). Specifically, we tested for signs of categorical encoding by examining whether reported orientations exhibited a staircase-like relationship with the actual target orientations—an expected signature of discretizing continuous information into categorical prototypes. The results instead showed a near-linear relationship, suggesting participants encoded and retrieved continuous orientation values with some variability, rather than mapping them onto discrete categories.

We also considered the possibility that participants may have used ensemble averaging strategies, encoding the mean orientation of all target items rather than each item individually. To evaluate this, we investigated whether reported orientations were systematically biased toward the mean orientation across targets within a trial. No such systematic biases were observed, and there was no evidence that the short presentation duration led to stronger attraction toward the mean. These findings suggest that participants did not rely on ensemble-based memory strategies, even under more challenging encoding conditions.

That said, we acknowledge that participants may have adopted various strategies to support encoding and maintenance (e.g., prioritization, grouping), and such strategies likely differ across individuals. However, in our design, all distraction conditions were randomly intermixed within subjects. This means that, although strategies may vary across participants, each individual likely applied a consistent strategy across conditions. Therefore, any strategy-based variability would not confound the within-subject contrasts that underlie our key findings. Future research could further examine how individual differences in memory capacity interact with strategic encoding and maintenance choices, particularly under high cognitive load.

Another noteworthy aspect of our continuous recall task is the use of a fixed initial orientation for the adjustable response bar in the probe display, which always pointed vertically upward. This design choice was consistent with our previous studies and aimed to minimize variability arising from random differences in the starting orientation of the response bar^36,38,39,65–69^. However, this fixed starting point may have introduced a potential concern: namely, that it could induce systematic biases in participants’ responses. To address this issue, we conducted a supplementary analysis examining the influence of the fixed initial orientation on response patterns (see Supplementary Materials, Figure S3-4, for details). The results showed that the fixed upward-pointing bar did introduce a minor attraction bias, primarily reflected in an increased proportion of unadjusted responses. Nevertheless, this bias was consistent across all trials and conditions, representing a systematic feature of the task rather than a source of random noise. Crucially, because all key experimental effects were assessed via within-subject comparisons between conditions, any such bias would have been held constant across conditions and thus is unlikely to confound the main findings reported in the present study.

While the current findings highlight how encoding duration interacts with distraction timing and task type to shape VWM performance, we acknowledge that the observed differences may not solely reflect variations in representational precision. In particular, the continuous recall and change detection tasks differ not only in the level of precision they require, but also in their response formats—recall versus recognition—which may engage different retrieval mechanisms. It is therefore possible that high-precision representations could be formed even under brief exposure durations, but their accessibility may depend on the nature of the memory test. Future studies could address this possibility by employing a change detection paradigm that systematically manipulates the precision requirements (e.g., by varying the similarity between targets and probes). Such designs would help disentangle the contribution of encoding quality from retrieval demands and provide a more nuanced understanding of how task structure shapes the manifestation of distraction effects in VWM.

## Conclusion

Across three experiments, our findings reveal a robust asymmetry in how distraction affects VWM across encoding and maintenance stages. Distractions occurring during the delay period consistently impaired memory performance, regardless of distractor type, perceptual load, or encoding duration—suggesting that once information is consolidated, it remains vulnerable to distraction from post-encoding inputs. In contrast, distraction during encoding produced more variable effects: it only impaired performance when encoding duration was brief and when task demands required high-fidelity memory representations. These results suggest that sufficient encoding time enables the formation of stable memory traces that can resist interference at early stages but not after consolidation is complete. Furthermore, we show that the content similarity between targets and distractors modulates distraction effects, particularly under longer encoding durations. While different-category distractors could often be suppressed, same-category distractors posed a greater challenge, especially when presented continuously or during the delay. Notably, increasing perceptual load did not amplify distraction costs, suggesting a stage-specific vulnerability of VWM that is not simply proportional to the amount of irrelevant information. Together, these findings underscore the critical role of presentation duration in shaping resistance to distraction and point to distinct mechanisms governing distractor suppression at different stages of VWM.

## Supplementary Information

Below is the link to the electronic supplementary material.

Supplementary Material 1

## Data Availability

Data that support the findings of this study have been deposited in the Open Science Framework at https://osf.io/65fa9/.
