# Supplementary material for: Early or late distractions hurt working memory differently depending on how long you look
Source: Sci Rep. 2025 Oct 9;15:35274. doi: 10.1038/s41598-025-18699-z (PMC12511591; doi:10.1038/s41598-025-18699-z)
Supplement: Supplementary file 1 — Supplementary Material 1 [file 41598_2025_18699_MOESM1_ESM.pdf]

Supplementary materials

Early or late distractions hurt working memory differently depending on how long you look

Guofang Ren [a], Ruyi Liu [b,c], Lijing Guo [a,b], Penglan Liu [b], Dan Nie [b], Jinru Chen [c], Chaoxiong Ye [a,b*]

a. School of Education, Anyang Normal University, Anyang 455000, China;

b. Department of Psychology, University of Jyvaskyla, Jyväskylä 40014, Finland;

c. Institute of Brain and Psychological Sciences, Sichuan Normal University, Chengdu 610066, China;

*** Correspondence to:** Chaoxiong Ye, E-mail: cxye1988@163.com

Cross-block accuracy in the change detection task of Experiment 1

To investigate the reviewer's concern regarding the potential impact of fatigue on participants' performance in the change detection task of Experiment 1, we conducted an exploratory analysis examining accuracy trends across the full duration of the task. Specifically, the 384 trials of the change detection task were divided into four consecutive blocks of equal length: Block 1 (trials 1–96), Block 2 (trials 97–192), Block 3 (trials 193–288), and Block 4 (trials 289–384). We then calculated the mean accuracy for each block and subjected these values to a one-way repeated-measures ANOVA with block (1–4) as the within-subject factor.

The analysis revealed a significant main effect of block on accuracy, $F(3,81) = 5.915$, $p = 0.002$, $\eta^2_p = 0.180$, indicating that performance systematically varied across the course of the task. Follow-up comparisons indicated that accuracy in Block 1 (0.714 ± 0.079) did not significantly differ from Block 2 (0.731 ± 0.070), $t(27) = 1.830$, $p = 0.078$, Cohen's $d = 0.346$, $BF_{10} = 0.864$. However, accuracy significantly improved in Block 3 (0.753 ± 0.080) compared to Block 1, $t(27) = 3.520$, $p = 0.002$, Cohen's $d = 0.665$, $BF_{10} = 22.937$, and in Block 4 (0.758 ± 0.089) compared to Block 1, $t(27) = 3.527$, $p = 0.002$, Cohen's $d = 0.667$, $BF_{10} = 23.311$. Block 4 also yielded significantly higher accuracy than Block 2, $t(27) = 2.610$, $p = 0.015$, Cohen's $d = 0.493$, $BF_{10} = 3.356$. No significant differences were found between Block 2 and Block 3, $t(27) = 1.833$, $p = 0.078$, Cohen's $d = 0.346$, $BF_{10} = 0.869$, or between Block 3 and Block 4, $t(27) = 0.333$, $p = 0.742$, Cohen's $d = 0.063$, $BF_{10} = 0.211$.

These results demonstrate a clear upward trajectory in performance over time, consistent with a practice effect rather than fatigue. Participants' accuracy improved gradually and reached a plateau toward the later blocks of the task. This pattern is commonly observed in visual working memory (VWM) paradigms, where participants often require several trials to establish consistent encoding and response strategies. Importantly, because all critical manipulations in our study (presentation duration and distraction condition) were implemented in a randomized within-subjects manner, this practice-related improvement is unlikely to confound the interpretation of the key effects reported in the main analyses.

In conclusion, the observed cross-block trend suggests that performance in the change detection task of Experiment 1 was not degraded by fatigue; rather, it improved with task experience. This finding supports the reliability of our main results and justifies their inclusion without further correction. The full dataset and analysis code for this supplementary analysis are available via the Open Science Framework at: https://osf.io/65fa9/.

Comparison of no-distraction condition performance between Experiment 1 and Experiment 2

To further examine whether the observed difference in accuracy of the change detection task between Experiment 1 and Experiment 2 might reflect a significant difference in baseline VWM performance, we conducted an additional between-experiment analysis focused on the no-distraction condition. This analysis aimed to test whether participants in the two experiments differed significantly in memory performance under matched baseline conditions.

We performed a 2 (Experiment: Experiment 1 vs. Experiment 2; between-subjects) × 2 (Presentation Time: short vs. long, based on the no-distraction condition; within-subjects) mixed-design ANOVA using accuracy in the no-distraction condition as the dependent variable. This design allowed us to assess potential main effects of experiment and presentation time, as well as their interaction.

The results revealed no significant main effect of Experiment, $F(1,53) = 2.212$, $p = 0.143$, $\eta^2_p = 0.040$, suggesting that overall baseline accuracy did not differ between participants in Experiment 1 and Experiment 2. There was also no significant main effect of Presentation Time, $F(1,53) = 2.976$, $p = 0.090$, $\eta^2_p = 0.053$. Furthermore, the interaction between Experiment and Presentation Time was not significant, $F(1,53) = 0.013$, $p = 0.910$, $\eta^2_p < 0.001$.

These results provide no evidence that baseline (i.e., no-distraction conditions) performance in the change detection task differed between Experiment 1 and Experiment 2. Thus, the pattern of differences observed in other experimental conditions is unlikely to be driven by differences in participants' baseline ability across experiments. Rather, the consistency in baseline performance supports the validity of interpreting the experimental manipulations (e.g., distraction condition and presentation duration) as the primary sources of variance in VWM performance across our experiments.

Pooled distraction cost analyses across experiments

To increase statistical power for analyzing distraction effects, we pooled data across experiments that included matched conditions within the same task type. For the change detection task, we combined data from Experiments 1 and 2, both of which included full-distraction and delay-distraction conditions. For the continuous recall task, we pooled data from Experiments 1 and 3, which shared identical encoding-distraction and delay-distraction conditions. For each task, we computed distraction costs by subtracting performance in each distraction condition from the corresponding no-distraction baseline, yielding distraction cost (accuracy) for the change detection task and distraction cost (offset) for the continuous recall task. These values were submitted to a two-way repeated-measures ANOVA with presentation duration (short vs. long) and distraction condition (encoding-distraction, full-distraction, delay-distraction) as within-subject factors. To further evaluate whether performance was significantly impaired under each condition, we conducted one-sample t-tests comparing distraction costs to zero. Partial eta squared ($\eta^2 p$) is reported for ANOVA effect sizes, and Cohen’s d and Bayes Factors (BF_{10}) are reported for the t-tests.

For the change detection task, the ANOVA on distraction cost (accuracy) revealed a significant main effect of the distraction condition, $F(2,54) = 20.168$, $p < 0.001$, $\eta^2_p = 0.428$. However, no significant main effect of the presentation time, $F(1,27) = 0.3$, $p = 0.589$, $\eta^2_p = 0.011$., and no significant interation was found between the presentation time and distraction condition, $F(2,54) = 0.72$, $p = 0.491$, $\eta^2_p = 0.026$.

We also conducted one-sample t-tests comparing the distraction cost (accuracy) in each condition to zero. Under the short presentation duration, the distraction cost in the encoding-distraction condition (-0.01036 ± 0.07753) did not differ significantly from zero, $t(27) = 0.707$, $p = 0.486$, Cohen's $d = 0.134$，$BF_{10} = 0.252$. In contrast, the distraction cost was significantly greater than zero in both the full-distraction condition (0.04873 ± 0.09029), $t(54) = 4.002$, $p < 0.001$, Cohen's $d = 0.54$，$BF_{10} = 122.6$, and the delay-distraction condition (0.05382 ± 0.08519), $t(54) = 5.073$, $p < 0.001$, Cohen's $d = 0.684$，$BF_{10} > 1000$. A similar pattern was observed under the long presentation duration. The distraction cost in the encoding-distraction condition (-0.002143 ± 0.08447) did not significantly differ from zero, $t(27) = 0.134$, $p = 0.894$, Cohen's $d = 0.025$，$BF_{10} = 0.202$. In contrast, the full-distraction condition (0.02909 ± 0.07372) showed a significant distraction cost, $t(54) = 2.927$, $p = 0.005$, Cohen's $d = 0.395$，$BF_{10} = 6.613$, as did the delay-distraction condition (0.06236 ± 0.09116), $t(54) = 4.685$, $p < 0.001$, Cohen's $d = 0.632$, $BF_{10} > 1000$. These findings confirm that only full-stage or delay-stage distractors reliably impaired performance in the change detection task, regardless of presentation time.

For the continuous recall task, the ANOVA on distraction cost (offset) revealed a

significant main effect of the distraction type, $F(2,54) = 6.747$, $p = 0.002$, $\eta^2_p = 0.2$. In contrast, there was no significant main effect of presentation duration, $F(1,27) = 0.009$, $p = 0.926$, $\eta^2_p = 0$, nor was there a significant interaction between presentation duration and distraction condition, $F(2,54) = 2.71$, $p = 0.076$, $\eta^2_p = 0.091$.

We also conducted one-sample t-tests to compare the distraction cost (offset) in each condition against zero. Under the short presentation duration, all distraction types showed significantly greater-than-zero distraction costs: encoding-distraction (3.02 ± 5.942), $t(51) = 3.665$, $p < 0.001$, Cohen's $d = 0.508$, $BF_{10} = 45.267$; full-distraction (3.224 ± 6.451), $t(27) = 2.644$, $p = 0.013$, Cohen's $d = 0.5$, $BF_{10} = 3.587$; and delay-distraction (3.7 4 ± 6.257), $t(51) = 6.232$, $p < 0.001$, Cohen's $d = 0.864$, $BF_{10} > 1000$. In contrast, under the long presentation duration, the distraction cost for encoding-distraction (1.188 ± 6.343) did not significantly differ from zero, $t(51) = 1.35$, $p = 0.183$, Cohen's $d = 0.187$, $BF_{10} = 0.355$. However, both full-distraction (2.792 ± 5.581), $t(27) = 2.648$, $p = 0.013$, Cohen's $d = 0.5$, $BF_{10} = 3.61$, and delay-distraction (6.9 ± 7.984), $t(51) = 4.31$, $p < 0.001$, Cohen's $d = 0.598$, $BF_{10} = 296.774$, yielded distraction costs that were significantly greater than zero. These results suggest that in continuous recall tasks, encoding-stage distraction is sensitive to the duration of visual input: when encoding time is short, participants are more susceptible to interference from concurrently presented distractors, whereas longer presentation durations allow for more complete consolidation and reduce vulnerability to such interference. However, distraction during the delay phase consistently disrupted memory performance regardless of encoding duration, further highlighting the limited resistance of consolidated VWM representations to post-encoding interference. Notably, unlike the pattern observed in the change detection task—where encoding-stage distraction had no reliable effect under either duration—continuous recall tasks appear to place greater demands on perceptual precision and thus reveal greater sensitivity to interference during early encoding stages.

Taken together, these pooled analyses provide converging evidence for two core conclusions. First, delay-phase distraction consistently impaired performance across both task types and encoding durations. This pattern was robust across measures (accuracy in change detection and offset in continuous recall), indicating that interference introduced after the encoding phase reliably degrades VWM performance, likely because it disrupts already-consolidated representations stored in a capacity-limited system.

Second, the impact of encoding-stage distraction differed substantially across task types. In the change detection task, encoding distraction was negligible regardless of presentation duration, suggesting that participants could effectively ignore concurrent distractors during encoding, even under brief exposure. In contrast, in the continuous recall task, encoding distraction significantly impaired memory under short exposure but had no effect under long exposure. This indicates that longer presentation times facilitated the consolidation of target representations and enabled more effective

filtering of concurrent distractors—an effect that was only detectable under the more sensitive demands of the continuous recall paradigm.

Overall, these findings suggest that the degree to which distraction impairs VWM depends both on when distraction occurs and on task-specific demands. While encoding-stage distraction can be mitigated through increased exposure or task structure, delay-stage distraction exerts a more persistent and general influence on memory performance, underscoring the fragile nature of consolidated visual information in the face of subsequent interference.

Testing for categorical encoding and ensemble-based strategy using in the continuous report task

To examine whether participants may have relied on categorical encoding strategies in the continuous report task, we conducted an analysis adapted from the Supplementary Materials by Zhang and Luck [1]. Specifically, we tested whether responses reflected coarse prototype-based representations (e.g., canonical orientations such as upward, rightward, or diagonal) rather than precise memory of the target orientation.

Data were pooled across all participants and conditions from Experiment 1. This pooling approach is justified, as orientation category boundaries tend to be highly consistent across individuals. For each trial, the reported orientation was plotted as a function of the actual target orientation (see Figure S1).

If participants encoded and retrieved the actual orientation with some degree of internal noise, the relationship between target and reported orientation should be approximately linear. In contrast, if categorical encoding strategies were used, the data would exhibit a staircase-like function, with plateaus corresponding to categorical bins and abrupt transitions at category boundaries.

As shown in Figure S1, the plotted data followed a smooth, continuous linear trend, with no visible signs of quantization or step-like shifts. This pattern suggests that participants encoded and reported precise orientation values rather than converting them into discrete categorical representations.

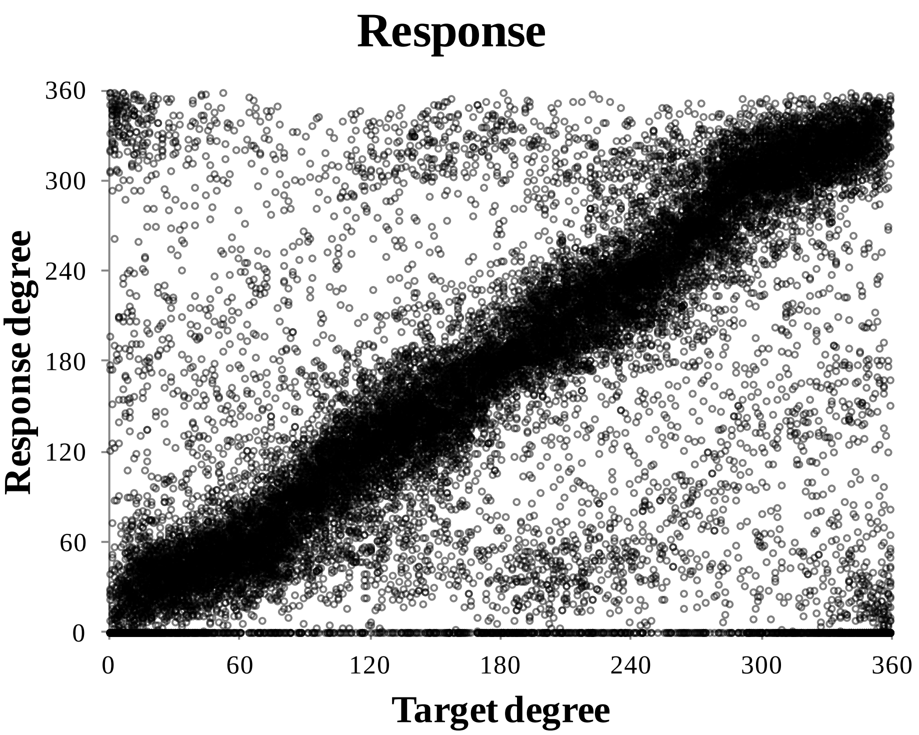

Figure S1. Scatterplot of reported orientation (y-axis) as a function of target orientation (x-axis), pooled across all participants and conditions in Experiment 1. Each dot represents a single trial. The diagonal linear trend indicates that participants' responses closely tracked the actual target orientations, consistent with analog encoding. No evidence of a staircase-like pattern—indicative of categorical encoding—was observed. This suggests that participants did not rely on coarse category prototypes but instead encoded and reported the true orientation values with some degree of noise.

In addition to testing for categorical encoding, we examined whether participants may have used an ensemble-based strategy—specifically, encoding the average of all target orientations—rather than storing each target individually. Such a strategy might be especially likely under more demanding conditions, such as the short presentation duration (200 ms), where encoding time is limited.

To test this possibility, we analyzed whether participants' responses in the continuous report task were systematically biased toward the mean orientation of the target items in each trial. If ensemble averaging occurred, we would expect reported orientations to be more strongly attracted toward the within-trial mean under short presentation durations compared to long durations.

Data were pooled across all participants and experimental conditions in Experiment 1, separately for the short and long presentation duration conditions. For each trial, we computed the mean orientation of the three target arrows and plotted the reported orientation as a function of this within-trial mean (see Figure S2).

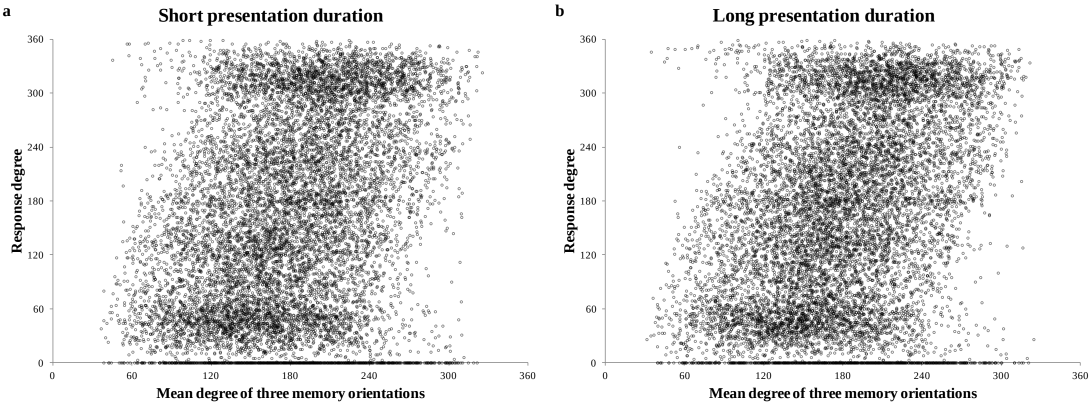

Figure S2. Scatterplots of reported orientation (y-axis) as a function of the mean orientation of the three memory targets (x-axis), shown separately for (a) short (200 ms) and (b) long (1000 ms) presentation durations in Experiment 1. Each dot represents a single trial pooled across all participants and conditions. If participants used ensemble-based strategies—such as averaging the target orientations—we would expect a positive linear relationship between the reported orientation and the mean of the memory array, especially under the short-duration condition where encoding demands are higher. However, no such relationship is observed in either duration condition, and no systematic differences emerge between panels a and b. These findings argue against the use of ensemble averaging as a dominant strategy for orientation encoding in this task.

If participants relied on ensemble averaging, we would expect to observe a linear trend in the scatterplots, indicating a systematic shift in reported orientations toward the mean of the target array. Moreover, the strength of this relationship should be stronger in the short presentation duration condition due to greater reliance on heuristic strategies under time pressure.

As shown in Figure S2, no clear linear relationship was observed between reported orientation and the within-trial mean orientation in either the short (Figure S2a) or long (Figure S2b) presentation condition. Furthermore, no systematic differences were found between the two duration conditions in terms of the dispersion or slope of the distributions. These results suggest that participants did not rely on ensemble averaging to guide their responses, even under encoding-constrained conditions.

Taken together, these analyses argue against the use of either categorical encoding or ensemble-based strategies as dominant approaches for representing multiple orientations in this task. Instead, participants appear to have attempted to maintain individual target representations in memory, even under brief encoding durations.

Analysis of systematic biases induced by the fixed probe orientation

In the continuous recall task of Experiment 1, the probe was always initialized at a fixed orientation of 0°, pointing vertically upward. Although this design ensured consistency across trials, it raised the possibility that the fixed probe might serve as an implicit visual cue, thereby biasing participants' memory reports either toward (attraction effect) or away from (repulsion effect) the 0° orientation.

To evaluate this possibility, we conducted a two-part analysis using the continuous recall data from Experiment 1. First, we tested for systematic directional biases in participants' signed error responses. Signed error was defined as the angular difference between the reported orientation and the true target orientation, with positive values indicating clockwise deviations and negative values indicating counterclockwise deviations.

To assess directional bias, we divided trials into two groups based on the angular location of the target: For targets between 1° and 179°, an attraction effect toward the 0° probe would manifest as a negative mean signed error, while a repulsion effect would yield a positive mean.

For targets between 181° and 359°, the pattern is reversed: a positive mean signed error would indicate attraction toward 0°, and a negative value would indicate repulsion.

One-sample t-tests comparing the mean signed error against zero showed: For the 1°–179° group, the mean signed error was significantly negative (−2.98° ± 6.72°), $t(27) = 2.35$, $p = .026$, Cohen's $d = 0.44$, $BF_{10} = 2.05$. For the 181°–359° group, the mean signed error (1.88° ± 5.86°) did not significantly differ from zero, $t(27) = 1.71$, $p = .099$, Cohen's $d = 0.32$, $BF_{10} = 0.72$. These results suggest a slight attraction bias toward the 0° probe orientation, particularly for targets on the 1°–179° half of the circle.

In the second part of the analysis, we examined the overall distribution of reported orientations across all trials (see Figure S3). Since the target orientations were uniformly randomized, the response frequencies across 1°–360° should be roughly uniform if no systematic preference exists. However, we observed a striking anomaly: participants selected the 0° orientation on 7.38% (±5.10%) of trials, significantly higher than the average selection probability for any other individual angle (0.26% ± 0.014%), $t(27) = 7.38$, $p < .001$, Cohen's $d = 1.40$, $BF_{10} > 1000$.

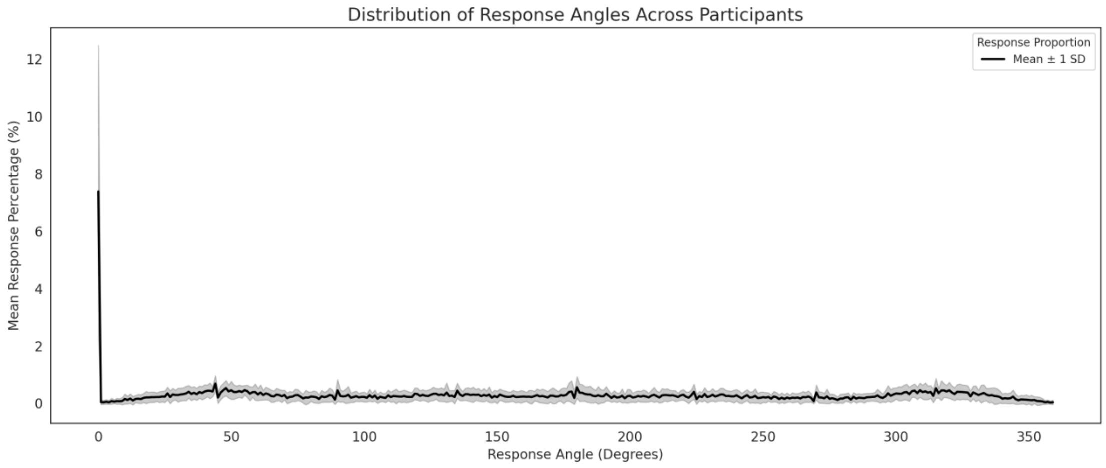

Figure S3. Mean response distribution across participants as a function of response angle (0°–359°). Each data point represents the average proportion of responses at each angle, aggregated across all participants. The shaded area denotes ±1 standard deviation, reflecting variability between participants. Response proportions were normalized within each participant before averaging. This distribution highlights potential angular biases or response tendencies, including a notable peak at 0°, where the probe was always initialized.

This overrepresentation of 0° responses suggests that in a substantial portion of trials, participants may have chosen not to adjust the probe from its default orientation. When trials with 0° responses were excluded, the response distribution across the remaining orientations was considerably more uniform (see Figure S4), indicating that the observed bias was primarily due to unadjusted responses.

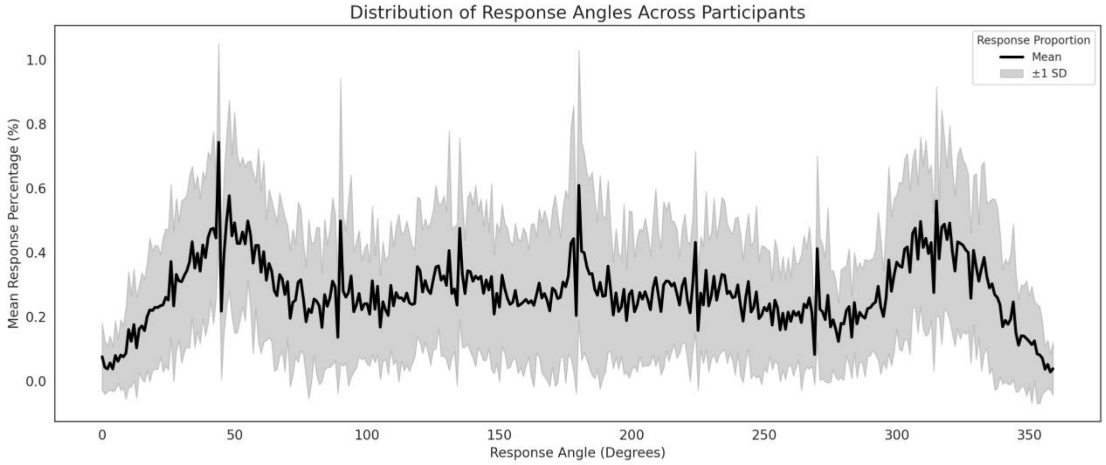

Figure S4. Mean response distribution across participants after excluding trials in which the response was exactly 0°. Each data point reflects the average normalized proportion of responses at each angle across participants. The shaded region indicates ±1 standard deviation, capturing between-subject variability. Compared to Figure S1, the distribution is notably more uniform, suggesting that the previously observed response bias was primarily driven by an overrepresentation of unadjusted 0° responses.

In summary, the fixed probe orientation at 0° appears to have induced a small but systematic attraction bias, mainly through the increased likelihood of participants retaining the default orientation. However, as this bias was consistent across conditions and participants, it constitutes a fixed procedural artifact and is unlikely to affect the validity of the within-subject contrasts that form the basis of our main findings.

Reference

1 Zhang, W. & Luck, S. J. Discrete fixed-resolution representations in visual working memory. *Nature* **453**, 233-235, doi:10.1038/nature06860 (2008).
